# *PIK3CA* Mutations: Are They a Relevant Target in Adult Diffuse Gliomas?

**DOI:** 10.3390/ijms26115276

**Published:** 2025-05-30

**Authors:** Ana Tomás, Marta Pojo

**Affiliations:** 1 Unidade de Investigação em Patobiologia Molecular (UIPM), Instituto Português de Lisboa Francisco Gentil (IPOLFG) E.P.E., 1099-023 Lisbon, Portugal; atomas@ipolisboa.min-saude.pt; 2 NOVA Medical School, NOVA University of Lisbon, 1169-056 Lisbon, Portugal

**Keywords:** glioma, glioblastoma, *PIK3CA* mutations, PI3K/Akt pathway, targeted therapy, PI3K inhibitors, alpelisib, cancer biomarkers

## Abstract

Gliomas are the most common and lethal malignant primary brain tumors in adults, associated with the highest number of years of potential life lost. The latest WHO classification for central nervous system tumors highlighted the need for new biomarkers of diagnosis, prognosis, and response to therapy. The PI3K/Akt signaling pathway is clearly implicated in tumorigenesis, being one of the most frequently altered pathways in cancer. Activating *PI3KCA* mutations are oncogenic and can influence both prognosis and treatment response in various tumor types. In gliomas, however, studies have reported inconsistent *PIK3CA* mutational frequencies, ranging from 0% to 30%. Furthermore, the impact of these alterations on glioma diagnosis, prognosis, and therapy response remains unclear. Current evidence suggests that *PIK3CA* mutations may represent early and constitutive events in glioma development, associated with worse glioblastoma prognoses, earlier recurrences, and widespread disease. Among these, the hotspot mutation H1047R has been particularly associated with a more aggressive phenotype while also modulating the neuronal microenvironment. In this review, we examine the clinical relevance of *PIK3CA* mutations across different cancers, with a particular focus on their emerging role in glioma. Moreover, we also discuss the therapeutic potential and challenges of targeting *PIK3CA* mutations in the context of glioma.

## 1. Introduction

Gliomas are the most common malignant primary brain tumors in adults (80–85%), representing ~23% of all primary central nervous system tumors [1,2]. Arising from the malignant transformation of glial cells, such as astrocytes and oligodendrocytes or their precursors [3,4,5], these tumors develop primarily in the brain, but can also appear in the spinal cord, brain stem, or cerebellum [2]. Diffuse gliomas are especially known to be highly heterogeneous, aggressive and infiltrative [6], being associated with approximately 20 years of potential life lost, the highest number out of most cancers [7]. Particularly, only ~7% of patients with glioblastoma multiforme (GBM), the most common and aggressive glioma subtype, survive five years after diagnosis [2]. The gold standard glioma treatment consists of maximal surgical resection followed by radiotherapy and chemotherapy, which is not efficient [8]. Even though these neoplasms do not typically metastasize to other organs, gliomas frequently invade surrounding brain tissue, presenting a diffuse phenotype. Thus, complete surgical resection becomes impossible, consequently leading to tumor recurrence and dismal prognosis [6,9]. Furthermore, due to the highly heterogeneous nature of these tumors, chemotherapy and radiotherapy resistance also occurs, preventing tumor cell elimination [10,11].

Until 2016, gliomas were classified into three main histological subtypes—oligodendroglial, astrocytic, and oligoastrocytic tumors [12,13]. This classification was based exclusively on histological criteria, by comparison of morphological characteristics between tumor cells and glial cells. Furthermore, a malignant grading system was applied, stratifying these entities on a scale of I, associated with lower anaplasia, low proliferation, and the most favorable prognosis, to IV, comprising often incurable, rapidly progressing, mitotically active neoplasms, with the highest degree of anaplasia [12]. Both histological classification and tumor grading were achieved using techniques such as light microscopy of hematoxylin and eosin-stained tumor sections and immunohistochemistry with different markers of lineage, proliferation, and differentiation, such as glial fibrillary acidic protein (GFAP), oligodendrocyte lineage transcription factor 2 (OLIG2), Ki67, and cytokeratin [12,14].

However, this approach to glioma stratification was flawed, and consequently, it was difficult to precisely evaluate tumors, diagnose patients, predict clinical outcomes, and find adequate therapies [15]. Molecular differences and the complexity among glioma subtypes became more and more evident as technological advancements were made, especially involving the genomic platforms for mRNA expression profiling and genome sequencing [16,17,18].

Hence, in 2016 the World Health Organization (WHO) incorporated isocitrate dehydrogenase (*IDH*) gene mutations and 1p/19q codeletion as essential biomarkers to adequately stratify gliomas, while also refining the previously established histological criteria [13]. The latest 2021 WHO classification of central nervous system tumors relies even further on these biomarkers to classify diffuse gliomas into three main types: *IDH*-wildtype GBM, *IDH*-mutant astrocytoma, and *IDH*-mutant and 1p/19q codeleted oligodendroglioma [19]. This update eliminates the previous diagnosis of *IDH*-mutant GBM, reclassifying it as astrocytoma, *IDH*-mutant, Grade 4. Consequently, all GBMs are now defined as *IDH*-wildtype. Other relevant molecular characteristics that aid in glioma diagnosis include epidermal growth factor receptor (*EGFR*) amplification, telomerase reverse transcriptase (*TERT*) promoter mutations or concurrent gain of chromosome 7 and loss of chromosome 10 in GBMs, and cyclin-dependent kinase inhibitor 2A/B (*CDKN2A*/*B*) homozygous deletion in *IDH*-mutant astrocytomas [19].

The introduction of these new biomarkers facilitated the prediction of patient outcome, as *IDH* mutations have been linked to favorable prognoses and sensitivity to chemotherapy [16,20,21,22]. Additionally, the 1p/19q codeletion has also been associated with better outcomes [23]. Overall, segregation among the established molecular glioma subgroups according to prognosis, diagnosis, and response to therapy became more accurate [16,22,24]. But even so, patient survival remains dismal, therapeutic strategies are still not efficient, and heterogeneity prevails in some molecular subgroups [6,16]. New molecular biomarkers are thus needed to improve glioma stratification and as therapeutic targets, to develop more efficient therapies.

Throughout the years, several molecular alterations have been proposed as biomarkers of prognosis or response to therapy, such as O-6-methylguanine-DNA methyltransferase (*MGMT*) promoter methylation, *EGFR* amplification, Phosphatase and Tensin Homologue (*PTEN*) deletion, and alpha thalassemia/mental retardation syndrome X-linked (*ATRX*) and *TERT* mutations [15,16,22,24]. Nonetheless, the clinical impact of these biomarkers has not been clear or coherent across multiple studies [16,25,26], and so research into new potential biomarkers in gliomas is still extremely relevant.

## 2. The Role of PI3K in Cell Signaling

Phosphatidylinositol-4,5-bisphosphate 3-kinase catalytic subunit alpha (*PIK3CA*) activating mutations have recently shown great promise as potential therapeutic targets in glioma [27,28,29]. *PIK3CA* is a 21-exon gene located on chromosome 3q26 that codes for the p110α subunit of Phosphatidylinositol-4,5-bisphosphate 3-kinase (PI3K) class I [30]. PI3Ks are a family of lipid kinases, acting as signal transducers in various pathways that are divided into three different classes according to function, structure, and substrate specificity—Class I, Class II, and Class III [31]. Class II and Class III PI3Ks have been less explored than Class I. Class II PI3Ks are monomeric kinases mainly involved in the regulation of membrane trafficking and angiogenesis through the production of phosphatidylinositol-3-phosphate (PI3P) and phosphatidylinositol-4,5-bisphosphate (PIP_2_) [32,33]. Class III PI3K consists of a single catalytic subunit, Vps34, and a regulatory subunit, Vps15, and can generate PI3P from phosphatidylinositol (PI) [34]. It plays an important role in autophagy regulation [34], but it is thought to also promote cell growth by interacting with the mechanistic target of rapamycin (mTOR) [35].

PI3K Class I, however, comprises some of the most well-studied kinases. These enzymes convert PIP_2_ to phosphatidylinositol-3,4,5-trisphosphate (PIP_3_) [30], and are divided into two subclasses, IA and IB, depending on the type of receptor to which PI3K binds. Class IB PI3Ks are activated by G-protein coupled receptors (GPCR) [31,36]. These are heterodimeric kinases, with a p110γ catalytic subunit and a p101 regulatory subunit [37]. On the other hand, Class IA PI3Ks are mostly activated by receptor tyrosine kinases (RTK). Specifically, Class IA has been the most studied so far in cancer research, since it promotes cell survival and proliferation [38]. These kinases are also heterodimeric, containing two distinct subunits: a catalytic subunit (p110α, p110β and p110δ, encoded by *PIK3CA*, *PIK3CB,* and *PIK3CD*, respectively) and a regulatory subunit (p85α, p85β and p55γ, encoded by *PIK3R1*, *PIK3R2,* and *PIK3R3*, respectively) [31]. Regulation and location can vary between all these kinases. For instance, p110δ is mainly expressed in leukocytes, being involved in immunity [39].

**Figure 1:**
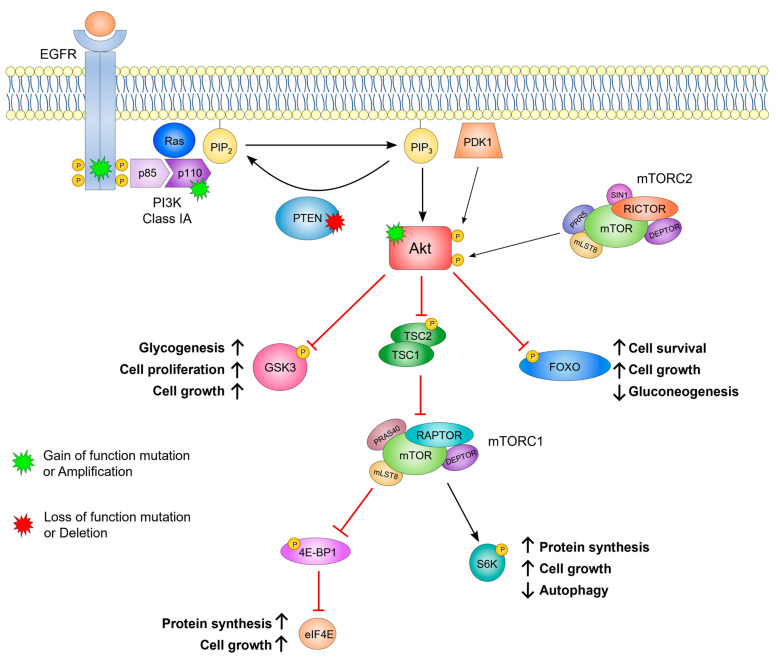
Overview of the PI3K/Akt signaling pathway. Upon ligand binding, receptor tyrosine kinases (e.g., EGFR) activate Class IA PI3K. Ras can act synergistically with EGFR to enhance PI3K activation. PI3K phosphorylates PIP_2_ to generate PIP_3_, which recruits PDK1 and Akt to the plasma membrane. PDK1 and mTORC2 phosphorylate Akt at Thr308 and Ser473, respectively, leading to its full activation. Activated Akt phosphorylates several downstream targets: GSK3 and FOXO are inhibited upon phosphorylation (blunt arrows), promoting biosynthesis, cell survival, growth, and proliferation (↑). TSC2 phosphorylation inhibits the TSC1-TSC2 complex, releasing its suppression of mTORC1. Activated mTORC1 phosphorylates S6K and 4E-BP1, further enhancing cell growth and protein synthesis (↑), while inhibiting autophagy (↓). PTEN negatively regulates this pathway by dephosphorylating PIP_3_ to PIP_2_. Key alterations in this pathway that promote tumorigenesis are highlighted in green and red. 4E-BP1, eukaryotic translation initiation factor 4E-binding protein; Akt, protein kinase B; EGFR, epidermal growth factor receptor; eIF4E, eukaryotic translation initiation factor 4E; FOXO, forkhead box O; GSK3, glycogen synthase 3; mTOR, mammalian target of rapamycin; mTORC1, mammalian target of rapamycin complex 1; mTORC2, mammalian target of rapamycin complex 2; PI3K, phosphatidylinositol-4,5-bisphosphate 3-kinase; PDK1, phosphatidylinositol-dependent kinase 1; PIP_2_, phosphatidylinositol-4,5-bisphosphate; PIP_3_, phosphatidylinositol-3,4,5-triphosphate; PTEN, phosphatase and tensin homologue; S6K, ribosomal protein S6 kinase; TSC1, tuberous sclerosis complex 1; TSC2, tuberous sclerosis complex 2.

Class IA PI3K is a key player in the PI3K/Protein Kinase B (Akt) pathway, a signaling pathway responsible for regulating a multitude of important biological processes, such as cell survival, proliferation, apoptosis, angiogenesis, and metabolism [31] (Figure 1). PI3K is activated when an RTK (e.g., EGFR) binds to a specific ligand, usually a growth factor [30]. Additionally, active GTPases from the Ras superfamily can directly bind to PI3K and promote its activation. Studies have shown that Ras acts synergistically with phosphorylated RTKs to enhance PI3K signaling [40]. PI3K phosphorylation then leads to the detachment of regulatory subunit p85 from catalytic subunit p110 and consequently to the release of catalytic inhibition, making it possible to modulate downstream signaling [41]. Thus, PI3K can phosphorylate PIP_2_ to PIP_3_, which in turn recruits serine/threonine kinase Akt to the cell membrane, one of the first steps in its activation [42]. To be fully active, Akt needs to be phosphorylated at serine and threonine residues. Phosphatidylinositol-Dependent Kinase 1 (PDK1) is a kinase that is also recruited by the second messenger PIP_3_, responsible for Akt phosphorylation at threonine residue Thr308 [43,44]. The mechanistic target of rapamycin complex 2 (mTORC2) is responsible for Akt phosphorylation at serine residue Ser473 [45]. Akt is, therefore, one of the leading players in this pathway, controlling multiple downstream targets. Glycogen synthase kinase 3 α and β (GSK3α/β) is one of those targets, becoming inactivated upon phosphorylation by Akt, ultimately leading to an increase in glycogen synthesis and cell proliferation [31,46]. Akt also inhibits Forkhead box O (FOXO) transcription factors, culminating in the inhibition of apoptosis, cell cycle arrest, and catabolism [31,47]. Additionally, Akt releases mTORC1 inhibition by the tuberous sclerosis complex 1 (TSC1)-TSC2 system when it phosphorylates TSC2. In turn, mTOR can regulate several downstream targets, including the activation of ribosomal protein S6 kinase (S6K) and inactivation of the repressor of mRNA translation eukaryotic translation initiation factor 4E-binding protein (4E-BP1), inducing biosynthesis and inhibiting autophagy (Figure 1) [48,49].

PTEN is considered the antagonist of this pathway, as well as a tumor suppressor, since it is a serine/threonine phosphatase that catalyzes the dephosphorylation of PIP_3_ to PIP_2_ [50].

Understanding the real complexities of this pathway is quite challenging, especially because it intertwines with several other signaling pathways, such as mitogen-activated protein kinase (MAPK)/extracellular signal-regulated kinase (ERK) [51,52], 5′ adenosine monophosphate-activated protein kinase (AMPK) [53], WNT/β-catenin [54], and transforming growth factor β (TGFβ) [55]. Regarding the role of this pathway in cancer, there are added degrees of complexity that we must consider. Innumerable genetic alterations that lead to PI3K/Akt constitutive activation, promoting proliferative self-sufficiency, might occur throughout these signaling cascades.

## 3. *PIK3CA* Gene Alterations

The PI3K/Akt pathway is one of the most frequently altered pathways in cancer, usually due to genomic alterations in *EGFR*, *PIK3CA*, *AKT*, and *PTEN* [56,57,58,59]. In a 2019 study that analyzed 60,991 solid tumor samples using next-generation sequencing (NGS), 44% of the samples analyzed harbored at least one molecular alteration (base-pair substitutions, insertions/deletions, copy-number alterations, and rearrangements) in one of 18 PI3K-related genes [56]. These alterations, considered pathogenic or likely pathogenic, potentially lead to the constitutive activation of the pathway, whether through overactivation of proteins that promote PI3K/Akt signaling or loss of its antagonist, inducing profound changes in intricate signaling networks, particularly involving cell survival, metabolism, and proliferation [58].

*PIK3CA* activating mutations are among the most common PI3K/Akt pathway alterations in cancer [56,57,60]. These are oncogenic alterations that, both in vitro and in vivo, promote cell invasion, proliferation, and angiogenesis while inhibiting apoptosis independently from upstream signaling [61,62,63,64].

**Figure 2:**
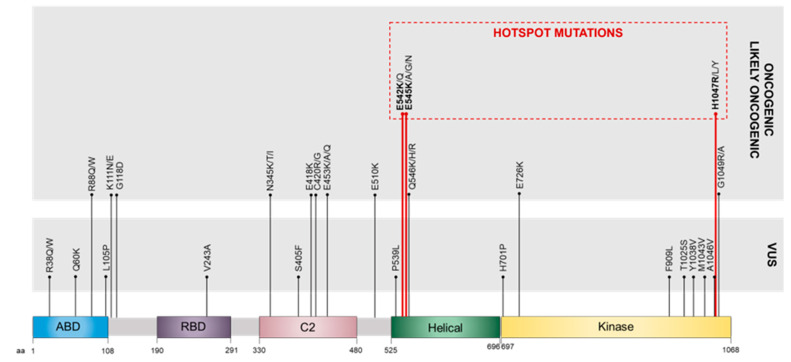
Main *PIK3CA* gene alterations reported in cancer. All five p110α domains are represented in a simplified schematic, along with examples of missense *PIK3CA* gene variants. These most commonly occur at the helical and kinase domains, where the main hotspot mutations, E542K, E545K, and H1047R, are in bold red. All variants are divided into oncogenic or likely oncogenic mutations and variants of uncertain significance (VUS). ABD, adaptor-binding domain; RBD, Ras-binding domain.

Catalytic subunit p110α, encoded by *PIK3CA*, has five distinct domains: the adaptor-binding domain (ABD), Ras-binding domain (RBD), C2 domain, helical domain, and kinase domain (Figure 2) [38]. *PIK3CA* mutations are found mainly at two of these domains: the helical domain (hotspot missense mutations E542K and E545K, located in exon 10) and the kinase domain (hotspot missense mutation H1047R, located in exon 21) [60]. Exons 10 and 21 of *PIK3CA*, according to Ensembl Transcript ID: ENST00000263967.4, RefSeq: NM_006218.4, are usually designated as coding exons 9 and 20, respectively, since the entire exon 1 is an untranslated region. Mutations in both of these hotspots can lead to oncogenic transformation by enhancing PI3K activity via one of two very distinct mechanisms [65]. H1047R blocks auto-inhibition by the C-terminal tail of the protein while also enhancing plasma membrane attachment. Furthermore, the amino acid substitution affects the ATP-binding site, making it more exposed and facilitating ATP binding [66]. Binding to regulatory subunit p85α is indispensable for the gain of function of kinase domain mutations, which are Ras-independent. On the other hand, E542K and E545K mutations prevent the binding of the p110α helical domain to the regulatory subunit p85α, being dependent on the interaction with Ras [65,67].

Alterations in the other PI3K domains, though less common, can still impact the protein’s function. ABD, for instance, interacts with the kinase domain within the protein’s three-dimensional structure, and mutations in this region may induce conformational changes in the kinase domain, thereby altering its enzymatic activity [68]. Additionally, mutations in the ABD or C2 domain can destabilize binding with the regulatory subunit, potentially leading to its disengagement and subsequent constitutive kinase activation [69]. The C2 domain, also important for PI3K’s association with the membrane, where its substrate PIP_2_ resides, may also undergo mutations that increase membrane affinity and enhance enzymatic activity [70,71]. Notably, RBD alterations are rarely reported, suggesting they are uncommon and that their impact on Ras binding to PI3K and subsequent oncogenicity potential remains unknown.

*PIK3CA* mutations in cancer were first reported by Samuels and colleagues in 2004 in a small cohort of 35 colorectal cancer patients [60]. This initial discovery was expanded to include multiple cancer types, such as GBMs, gastric, breast, and lung cancers, leading to the recognition of *PIK3CA* as a key oncogene. Since then, extensive research has been directed toward understanding the frequency of *PIK3CA* mutations across different cancer types and their clinical implications on prognosis and response to therapy.

The PI3K/Akt pathway is particularly relevant in breast cancer, where *PIK3CA* alterations occur in roughly 21% to 47% of cases [56,59,72,73,74,75,76,77,78,79,80,81,82], making it the most frequently mutated oncogene in this cancer type. These mutations are predominantly found in hormone receptor (HR)-positive and human epidermal growth factor receptor 2 (HER2)-positive breast cancers [79,81,83,84]. However, their prognostic impact in breast cancer remains unclear. Some studies report *PIK3CA* mutations as independent negative prognostic factors [76,85] while others associate them with improved patient outcomes [75] and some find no significant correlation [59,78,79,81]. These findings suggest a context-dependent impact, likely influenced by breast cancer subtypes, and with specific mutations, such as the hotspot H1047R, linked to more aggressive disease and poorer prognoses compared to E345K [74,86,87,88]. Additionally, *PIK3CA* hotspot mutations may also contribute to therapy resistance in breast cancer, with associations found between these mutations and resistance to chemotherapy (e.g., paclitaxel, anthracyclines) [64,88,89] and HER2-targeted therapies like trastuzumab [82,90,91,92].

These mutations have also shown to be quite relevant in colorectal cancer, present in about 10% to 32% of tumors [56,60,93,94,95,96,97,98,99,100,101], and are often associated with worse prognoses [100,101,102]. However, there are some contradicting studies that do indicate no significant impact on clinical outcomes or tumor progression [94,95,96,97,98,103]. *PIK3CA* mutations are also implicated in acquired resistance to anti-EGFR therapy in metastatic colorectal cancer patients [101,104], similar to the resistance to trastuzumab seen in breast cancer. This aligns with the role of EGFR and HER2 in PI3K/Akt signaling, where downstream *PIK3CA* mutations can override inhibition of the RTKs targeted by these therapies. Additionally, these mutations seem to also be associated with chemoresistance in colorectal cancer [99].

Alterations in *PIK3CA* are also present in gastric (2–25%) [56,60,105,106,107,108,109,110,111], endometrial (16–51%) [112,113,114,115], head and neck (6–21%) [56,116,117,118,119,120], and esophageal cancers (5–21%) [121,122,123,124,125,126]. In gastric and endometrial cancers, these mutations are generally not significantly associated with patient prognosis [105,106,107,108,109,110,113,114,127]. However, specific exon 10 mutations have been linked to poorer outcomes in endometrial cancer [115], and a meta-analysis reported that *PIK3CA* mutations negatively impact survival in these patients [128]. Notably, while these mutations are not considered prognostic markers in gastric cancer, they have been associated with increased tumor aggressiveness [109]. In contrast, in esophageal cancer, *PIK3CA* mutations are regarded as independent favorable prognostic factors [121,122,123].

Given the complexity of the PI3K pathway within the broader network of signaling cascades, it is expected that *PIK3CA* mutations may exert paradoxical effects in different oncogenic contexts, even within the same type of cancer. Not only that, but the observed variability in *PIK3CA* mutational frequencies and prognostic associations across many studies is likely influenced by differences in methodology (e.g., Sanger sequencing versus NGS), the specific exons analyzed (e.g., only hotspot exons 10 and 21 versus all coding exons), and the diversity of patient cohorts.

## 4. *PIK3CA* Mutations as Biomarkers in Glioma

### 4.1. Mutation Frequency

The frequency and impact of oncogenic *PIK3CA* mutations has also been explored in glioma, yet questions remain about their true relevance in this context. Studies report a wide range of mutation frequencies in GBM, from 0% to 30% [18,27,28,60,129,130,131,132,133,134,135,136] (Table 1). In their pioneer study, Samuels et al. reported a 27% *PIK3CA* mutation frequency in a small GBM cohort (*n* = 15) using Sanger sequencing. However, they may have overestimated mutation prevalence due to the limited sample size [60]. Gallia et al. also analyzed a small cohort of 38 primary GBM cases, reporting a 17% mutation rate, though they excluded some *PIK3CA* exons, potentially underrepresenting true mutation rates [130]. In contrast, larger cohorts often yield lower frequencies. Broderick and colleagues observed only a 5% mutation frequency in a GBM cohort of 105 samples, albeit analyzing only exons 10 and 21 [129]. However, similar frequencies (5–7%) were obtained by other authors, using different detection methods and sample sizes (from 70 to 116 samples). These studies managed to analyze more *PIK3CA* exons, with some even being able to sequence all coding exons [131,132,133,134]. Of note, even studies covering all coding exons have yielded inconsistent findings, with one study identifying no *PIK3CA* mutations in 30 GBM samples [135].

Recent advances, such as NGS, have enabled a more comprehensive coverage, revealing a *PIK3CA* mutation frequency of 10–11% in larger cohorts (*n* = 130 [27], and 291 [18], respectively), though rates as high as 30% have been reported in certain multifocal GBM cases [27]. Tanaka et al., using a multiplex detection system focused on six known *PIK3CA* hotspots (R88, E542, E545, Q546, H1047, G1049), found an 8.3% *PIK3CA* mutation rate in 157 GBM samples [28]. On the other hand, Saadeh et al. reported a higher frequency of 21.7% using whole exome sequencing on a smaller cohort of 60 GBM [136].

Research regarding *PIK3CA* mutation frequencies in lower-grade glioma has been similarly inconclusive, with mutation rates reported from 0% to 14% depending on the histological subtype and methodology used [129,134,137] (Table 1). Broderick et al. analyzed the hotspot *PIK3CA* exons 10 and 21 and identified a 3% mutation rate in 31 astrocytomas and a higher 14% frequency in 21 oligodendrogliomas [129]. Hartmann et al. reported a lower *PIK3CA* mutation rate of 5% in oligodendrogliomas in a slightly bigger cohort (*n* = 66) by analyzing exons 2, 10, and 21 [137]. On the other hand, more recently Wang et al. analyzed all coding exons and reported a 9.6% mutation rate in 52 astrocytomas but found no *PIK3CA* mutations in a small subset of 17 oligodendrogliomas [134].

These notable inconsistencies across studies stem from differences in sample sizes, mutation detection methods, exon coverage, and outdated histological classification systems. Furthermore, the presence of mutations in a pseudogene with over 95% sequence homology to *PIK3CA* exon 10 may also confound these results [140,141], which none of the studies above mentioned.

Although several studies have examined the frequency of *PIK3CA* mutations in glioma, most were mainly based on outdated histological classifications, with few considering the updated molecularly defined subgroups. To date, only one study has investigated *PIK3CA* mutation rates across all glioma molecular subtypes defined by the 2016 WHO classification, using a large cohort of 394 adult diffuse gliomas, albeit only analyzing exons 10 and 21 [29]. Mutation frequencies ranged from 3% to 10%, with higher rates in 1p/19q codeleted and *IDH*-mutant oligodendrogliomas and *IDH*-wildtype astrocytomas (10%), and lower frequencies in GBM *IDH*-wildtype (3%), the most aggressive glioma subtype. Notably, similar frequencies were found in The Cancer Genome Atlas (TCGA) dataset—3% versus 2% in GBM *IDH*-wildtype, and 9% versus 8% in the now obsolete subgroup of GBM *IDH*-mutant. Compared with the findings reported by TCGA in 2015 [138] and Dono and colleagues [139], the observed *PIK3CA* mutation frequency in 1p/19q codeleted and *IDH*-mutant gliomas was lower in this cohort (10% versus 20% and 14%, respectively) [29], likely due to the more limited sequencing strategy. Thus far, no studies have addressed *PIK3CA* mutation frequency across the glioma subgroups defined by the 2021 WHO classification. As such, much remains to be explored regarding the potential of *PIK3CA* mutations as biomarkers in glioma.

### 4.2. Clinical and Biological Impact

While most studies have focused primarily on *PIK3CA* mutational frequencies, there has been limited research into their impact on prognosis and therapy response in glioma, which remains poorly understood. Moreover, research in this field appears significantly outdated—studies exploring the role of *PIK3CA* mutations considering the most recent glioma molecular stratification are severely lacking.

Regarding the impact of *PIK3CA* mutations on GBM patient outcomes, an association with poor prognoses has been reported [27,28]. Lee J. and colleagues demonstrated that patients with GBM harboring *PIK3CA* mutations had worse prognoses than wildtype patients, although no multivariable analysis was conducted [27]. Another study linked *PIK3CA* mutations to reduced progression-free survival of patients with GBM *IDH1*-wildtype, independent of other variables [28]. Additionally, by studying the imaging characteristics of *PIK3CA* mutant GBM, this same study found a significant association between these mutations and widespread disease at diagnosis, compatible with gliomatosis, multicentric lesions, or distant leptomeningeal lesions. However, no association with overall survival was found, consistent with the findings of Brito et al. [29], who showed that *PIK3CA* alterations were not independent prognostic factors in GBM *IDH*-wildtype, despite a trend toward shorter survival.

Beyond their potential prognostic relevance in GBM, *PIK3CA* mutations have also been described as early events in GBM development, found in all sectors of the tumor [27]. Moreover, analyses of matched primary and recurrent tumor samples have shown that these mutations constitute early events that are maintained through glioma progression, regardless of the therapy administered [29]. These findings suggest that *PIK3CA* mutations may represent a stable and therapeutically actionable target in these highly heterogenous tumors, while also hinting at a possible role in therapy resistance.

In lower-grade gliomas, the prognostic value of *PIK3CA* mutations has been explored to a limited extent. Only two studies to date have assessed the impact of these mutations on patient survival in this context. Wang and colleagues [134] reported that patients with *PIK3CA*-mutated lower-grade gliomas had significantly lower overall and progression-free survival than patients with wildtype tumors. However, the impact on patient prognosis was not independent from other variables, and the number of *PIK3CA*-mutated samples was low (5/69). Regarding *IDH*-mutant and 1p/19q-codeleted oligodendrogliomas, Dono et al. showed that *PIK3CA* mutations are associated with worse overall survival in a multivariable analysis [139].

While clinical data on the prognostic relevance of *PIK3CA* mutations in glioma continues to grow, research into their underlying pathogenic mechanisms remains scarce. McNeill and colleagues demonstrated that *PIK3CA* mutations promote astrocyte growth by potentiating PI3K signaling [142]. Interestingly, only the H1047R mutation induced anchorage-independent colony formation. Nevertheless, both helical and kinase domain mutations potentiated RAS-mutant astrocyte tumorigenesis in vivo. Similarly, Tateishi et al. showed that *PIK3CA* mutations seem to drive oligodendroglioma progression in an orthotopic mouse model [143]. Furthermore, another in vivo study identified H1047R and C420R as key players in neuronal microenvironment modulation [144], suggesting they are main drivers of gliomagenesis, acting through different mechanisms. However, most clinical studies overlook the differential impact of specific mutations like H1047R or E545K on patient outcomes, representing a gap in the current literature.

Overall, studies point to a potential important role of *PIK3CA* mutations in glioma, but the current body of evidence remains inconsistent. Robust multicentric studies, using the most recent 2021 WHO classification, are needed to clarify the role of these mutations in gliomagenesis, aggressiveness, prognosis, and therapy response. In this case, the acquisition of enough samples of each subgroup for a robust analysis might be a challenge, especially as all evidence points to *PIK3CA* mutations not being too abundant in glioma. Given the heterogeneous genetic landscape of gliomas, these mutations may ultimately be one of many within the signaling cascade, potentially lacking unique prognostic value without stratified, subtype-specific analysis. Additionally, examining how *PIK3CA* mutations may modulate treatment response, particularly to temozolomide, is critical, given the current evidence of these mutations conferring resistance to multiple therapeutic strategies in other cancers [64,82,88,89,90,91,92,99,101,104].

With glioma remaining an aggressive and virtually incurable type of cancer, understanding its biology is vital to identify effective therapies. Thus, the use of *PIK3CA* mutations as actionable targets in glioma should be explored further.

## 5. Could PI3Kα Inhibitors Offer a New Approach to Target Glioma?

Knowing that alterations in the PI3K/Akt pathway are so common and important in cancer, multiple antagonists have been developed throughout the years in the hopes of providing better and more efficient therapeutic strategies to cancer patients, especially as so many of them harbor upregulation of this pathway and often develop resistance to standard treatment [38].

**Figure 3:**
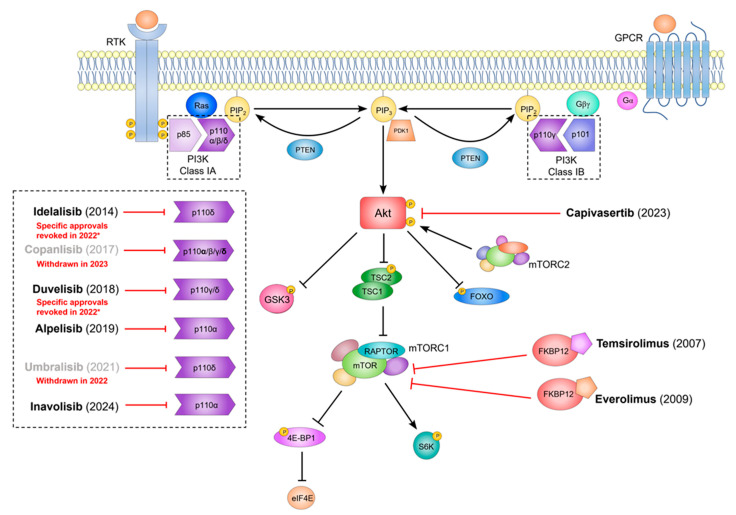
Inhibitors targeting the PI3K/Akt pathway, clinically approved for cancer treatment. PI3K, Akt, or mTOR inhibitors aim to reduce cell proliferation, growth, and biosynthesis, and are indicated by the red blunt-end arrows. Temsirolimus (approved in 2007 by the FDA) and everolimus (2009) bind to FKBP12, inhibiting mTORC1, and are used mainly in advanced renal and breast cancers. Capivasertib, an Akt inhibitor, was approved in 2023 for use in the treatment of HR-positive, HER2-negative breast cancer. Five PI3K inhibitors have been approved for clinical use. Idelalisib (2014), umbralisib (2021), and duvelisib (2018) target p110δ or p110 γ/δ PI3K catalytic subunits, while copanlisib (2017) is a pan-PI3K inhibitor with predominant activity against p110α and p110δ. These inhibitors were approved for use in hematological cancers but faced safety-related withdrawals or restrictions: umbralisib and copanlisib were withdrawn from the market in 2022 and 2023, respectively, while idelalisib and duvelisib had specific approvals revoked and their use has been restricted (*). In contrast, the selective p110α inhibitors alpelisib and inavolisib were approved in 2019 and 2024, respectively, for the treatment of advanced HR-positive, *PIK3CA*-mutant breast cancer, alpelisib in combination with fulvestrant and inavolisib in combination with palbociclib and fulvestrant. FDA, U.S. Food and Drug Administration; FKBP12, FK506 binding protein-12; GPCR, G protein-coupled receptor; RTK, receptor tyrosine kinase.

Currently, a plethora of inhibitors targeting this pathway is being researched and undergoing clinical trials [145]. So far, nine inhibitors targeting the PI3K/Akt/mTOR pathway have already been clinically approved by the U.S. Food and Drug Administration (FDA) for use in cancer treatment in specific contexts (Figure 3): temsirolimus and everolimus (mTOR inhibitors) [146,147], capivasertib (AKT inhibitor) [148], idelalisib and umbralisib (both PI3K inhibitors, selective for catalytic subunit p110δ) [149,150], copanlisib (pan-PI3K inhibitor targeting all four class I PI3Ks, with a preference for PI3Kα and PI3Kδ) [151], duvelisib (dual-PI3Kδ/PI3Kγ inhibitor) [152], and alpelisib and inavosilib (selective for catalytic subunit p110α) [153,154].

However, safety concerns have emerged regarding four out of the six PI3K inhibitors, which were approved for use in hematological malignancies given the enrichment of p110δ and p110γ in leukocytes. Umbralisib and copanlisib were both withdrawn after recent clinical trials failed to confirm clinical benefit and raised safety issues. Similarly, idelalisib had its approval for use in small lymphocytic lymphoma (SLL) and follicular lymphoma (FL) withdrawn and now faces restricted use in chronic lymphocytic leukemia (CLL) due to toxicity concerns. Duvelisib is approved for use in CLL and SLL but had its FL approval withdrawn. Many clinical trials based on these four inhibitors have been terminated. Consequently, new-generation PI3K inhibitors are under investigation for hematological malignancies with an emphasis on enhancing tolerability [155,156,157]. Still, in other oncological contexts, PI3K inhibition has shown great promise (summary in Table 2).

Notably, several studies have reported *PIK3CA* mutations as predictive biomarkers of response to PI3K/Akt pathway inhibitors [120,158,172,173,174]. However, concerns have emerged regarding the use of *PIK3CA* status alone to guide therapeutic decisions. Indeed, it seems that increased sensitivity to PI3K/Akt pathway inhibitors is not universal to all types of cancer or to all classes of inhibitors, and the presence of different *PIK3CA* mutations might lead to distinct inhibitor sensitivities [161,168,170,177,182,185,192,193,205,206,207]. Moreover, there is still a scarcity of clinical trials explicitly designed to evaluate how *PIK3CA* mutations influence response to PI3K/Akt pathway inhibitor in different types of cancer.

In general, pan-PI3K inhibitors have shown limited efficacy in cancer therapy due to increased toxicity when compared with more targeted approaches [58,198,202]. Furthermore, efficiency is usually very limited since patients often acquire resistance to these inhibitors, either due to acquired mutations in regulatory genes or the activation of compensatory mechanisms and pathways. As such, the field has shifted toward isoform-selective PI3K inhibitors, which tend to exhibit better efficacy with fewer off-target effects [208].

Alpelisib (BYL719) is an orthosteric p110α selective isoform inhibitor from the 2-aminothiazole family, which blocks catalytic activity by binding to the ATP-binding site [209]. Researchers have shown particular interest in studying the response to this inhibitor in the presence of *PIK3CA* mutations, seeing as it is highly specific toward PI3Kα and so it is expected to reduce off-target toxicity [208]. Indeed, alpelisib has a more pronounced antitumor effect in the presence of *PIK3CA* mutations, which is amplified in the presence of double mutations [161]. In 2019, it was approved for clinical use in patients with advanced HR-positive and *PIK3CA*-mutant breast cancer, in combination with fulvestrant, a selective estrogen receptor degrader [153,158,159]. More recently, in 2024, inavolisib (GDC-0077)—another selective orthosteric PI3Kα inhibitor that additionally promotes mutant PI3Kα degradation [185]—emerged as an additional treatment option for HR-positive, HER2-negative, *PIK3CA*-mutant breast cancer, in combination with palbociclib, a CDK4/6 inhibitor, and fulvestrant [154,183].

Nevertheless, as with other targeted therapies, p110α selective isoform inhibitors do not always reach their full potential. One major limitation is the activation of compensatory signaling pathways, which can undermine treatment efficacy and lead to drug resistance [210,211]. Resistance mechanisms include upregulation of insulin, PIM kinases, IGF1R, PI3Kβ, and mTOR signaling, as well as PTEN loss and secondary *PIK3CA* mutations [206,212,213,214,215,216]. In HER2-positive, *PIK3CA*-mutant breast cancer, for instance, HER3 upregulation via HER2-induced phosphorylation is a key compensating mechanism [210,217]. Accordingly, combination strategies that co-target these alternative pathways or with already existing standard-of-care treatments can enhance the efficacy of PI3Kα inhibitors [145,171,199,218,219].

Emerging evidence suggests that secondary *PIK3CA* mutations may confer resistance to orthosteric inhibitors, such as alpelisib, which might be overcome by AKT inhibition or novel allosteric selective PI3Kα inhibitors [206]. Notably, a new mutant-selective allosteric PI3Kα inhibitor, RLY-2608, has shown great promise in circumventing some of the downfalls associated with orthosteric inhibitors, including toxicities related with glucose homeostasis and the skin [193].

In gliomas, however, the role of *PIK3CA* mutations in modulating sensitivity to PI3K pathway inhibition remains poorly understood. Buparlisib, a brain-penetrant pan-PI3K inhibitor with preferential activity against PI3Kα, is the most extensively studied PI3K inhibitor in GBM (Table 2). Nevertheless, it has shown limited efficacy in clinical trials, whether used as monotherapy [177] or in combination with other agents [178]. Of note, *PIK3CA* mutational status was not assessed in the latter study, and preclinical data suggests that specific *PIK3CA* mutations may modulate response to buparlisib when in combination with MEK inhibitors [142]. Therefore, it remains unknown whether combination therapy would be more efficient in the presence of *PIK3CA* mutations. Other brain-penetrant inhibitors such as paxalisib (GDC-0084) and voxtalisib (XL765), both dual PI3K/mTOR inhibitors, have shown more favorable outcomes in preclinical and clinical studies [186,187,188,189,199,200]. However, the predictive role of *PIK3CA* mutations was not assessed.

Regarding alpelisib, the p110α selective inhibitor that has shown such promise in *PIK3CA*-mutant breast cancer, less research has been conducted in the context of gliomas. Preclinical studies show that alpelisib combined with other PI3K/Akt pathway inhibitors more effectively reduces Akt phosphorylation in PTEN-null GBM models than alpelisib alone, suggesting that PTEN-null GBM cells can rely on both PI3Kα and PI3Kβ isoforms to maintain cell survival [162,163]. These findings are extremely relevant in the context of glioma, since PTEN loss is quite frequent in GBM [220,221]. Similar compensatory mechanisms have been described in other types of cancer, such as breast, colon, head and neck, and prostate, where PTEN-deficient tumors sustain proliferation through PI3Kβ and are thus most likely not to respond to PI3Kα inhibition [191,214,216,222].

Still, none of these studies have analyzed the effect of PI3Kα selective isoform inhibitors specifically in *PIK3CA*-mutated glioma. While alpelisib shows a clear clinical benefit in *PIK3CA*-mutant breast cancer, the complexity of signaling networks in glioma may require broader or combination-based strategies. Paradoxically, pan-PI3K inhibitors, though less favored in other types of cancer, may offer better coverage in glioma if used alongside the targeting of other signaling pathways, as shown in Table 2. Furthermore, the relationship between PTEN deficiency and *PIK3CA* mutations and how it affects alpelisib treatment response in glioma must be further explored. Curiously, in an ovarian cancer cell line harboring both PTEN loss-of-function and *PIK3CA* mutations, only the inhibition of the p110α isoform, and not p110β, resulted in cell growth suppression [222]. This finding seems to indicate that, in the presence of both alterations, *PIK3CA* mutations might triumph over PTEN loss-of-function, allowing the efficient targeting of the p110α isoform. Hence, understanding the connection between these alterations might be the key to the efficient targeting of the PI3K/Akt pathway in glioma.

## 6. Future Perspectives

Gliomas remain highly challenging and aggressive tumors, often recurring and causing significant patient morbidity and mortality. As such, identifying reliable biomarkers is imperative to better stratify these patients and develop new efficient therapies. Given the prominent role of the PI3K/Akt pathway in other cancers, where *PIK3CA* mutations sometimes serve as prognostic or predictive biomarkers, it is crucial to further investigate the real impact of these mutations on glioma pathogenesis, prognosis, and therapy response. Although recent studies have shed some light on the role of *PI3KCA* in glioma, our current understanding remains limited. Considering the updated 2021 WHO classification of central nervous system tumors, future research must aim to clarify the role of *PIK3CA* mutations across glioma molecular subtypes. Large, rigorous, multicentric studies involving extensive patient cohorts will be essential to determine the true impact of *PIK3CA* mutations within each subgroup. Advanced sequencing methods, including the generalized use of NGS, can facilitate a more comprehensive analysis of *PIK3CA* mutations, although assessment of the pseudogene with exon 10 homology must be prioritized for accurate results. These efforts could ultimately reveal whether *PIK3CA* mutations are genuine drivers of glioma pathogenesis or simply represent another layer of the extensive glioma molecular complexity.

The identification of *PIK3CA* mutations as early and constitutive events in glioma progression strongly hints that these alterations might be valuable therapeutic targets, even if they are relatively infrequent. Selective PI3Kα inhibition in *PIK3CA*-mutant low-grade gliomas could potentially suppress disease progression at an earlier stage. In GBM, the most prevalent and lethal glioma subtype, targeting PI3Kα could also serve as a promising therapeutic approach, particularly in multifocal cases where *PIK3CA* mutations are consistently present across all lesions. However, PI3Kα inhibitors, when used as monotherapies, often exhibit limited efficacy due to the presence of redundant resistance mechanisms, which should not be overlooked. Unlike in breast cancer, selective targeting of p110α in glioma may encounter challenges due to reliance on other PI3K isoforms, PTEN loss-of-function, and interactions with additional survival pathways. Therefore, investigating the interplay between *PIK3CA* mutations, p110β expression, PTEN loss, and other glioma-associated alterations is essential to refine PI3K-targeted approaches in glioma. Tailoring PI3K inhibition based on the tumor’s molecular profile could yield more precise and individualized treatments in glioma, rather than the more generalized approach that has been used in recent clinical trials. Furthermore, assessing whether PI3Kα inhibition could enhance glioma sensitivity to standard therapies like temozolomide and radiotherapy may also open new avenues for more effective combination treatment strategies.

## Figures and Tables

**Table 1:** Previous research on *PIK3CA* mutation frequency and clinical impact in glioma subgroups.

Classification	Glioma Subgroup	Sample Size	Method	Region Evaluated	Mutation Frequency	Prognostic Effect (U/M)	Reference
Histological	GBM	30 (Multifocal)	Illumina HiSeq	All coding exons	30%	Unfavorable (U)	[27]
		130 (Solitary)			10%		
		15	Sanger sequencing	All coding exons	27%	---	[60]
		60	Illumina HiSeq	All coding exons	21.7%	---	[136]
		38	Sanger sequencing	Exons 2, 3, 5, 6, 8, 10, 13, 14, 19 and 21 *	18%	---	[130]
		291	Illumina HiSeq	All coding exons	11%	---	[18]
		157	SNaPshot^®^ multiplex system	6 known hotspots	8.3%	Unfavorable (M)	[28]
		70	PCR-SSCP direct sequencing	All coding exons	7%	---	[132]
		116	Affymetrix microarray	All coding exons	6%	---	[131]
		105	Sanger sequencing	Exons 10 and 21 *	5%	---	[129]
		97	PCR-SSCP direct sequencing	Exons 2, 3, 5, 6, 8, 10, 13, 14, 19 and 21 *	5%	---	[133]
		40	Ion semiconductor sequencing	All coding exons	5%	---	[134]
		30	PCR-SSCP	All coding exons	0%	---	[135]
	Astrocytoma	52	Ion semiconductor sequencing	All coding exons	9.6%	Unfavorable (U)	[134]
		31	Sanger sequencing	Exons 10 and 21 *	3%	---	[129]
	Oligodendroglioma	21	Sanger sequencing	Exons 10 and 21 *	14%	---	[129]
		66	PCR-SSCP direct sequencing	Exons 2, 10 and 21 *	5%	---	[137]
		17	Ion semiconductor sequencing	All coding exons	0%	---	[134]
Molecular	GBM, *IDH*-wildtype	567 (TCGA)	Illumina HiSeq	Exons 10 and 21*	2%	Not significant (M)	[29]
		239	Sanger sequencing		3%		
	GBM, *IDH*-mutant	25 (TCGA)	Illumina HiSeq	Exons 10 and 21 *	8%	---	[29]
		11	Sanger sequencing		9%		
	Astrocytoma, *IDH*-wildtype	39	Sanger sequencing	Exons 10 and 21 *	10%	---	[29]
	Astrocytoma, *IDH*-mutant	56	Sanger sequencing	Exons 10 and 21*	5%	---	[29]
	1p/19q codeleted + *IDH*-mutant	84	Illumina HiSeq	All coding exons	20%	---	[138]
		107	Illumina HiSeq	All coding exons	14%	Unfavorable (M)	[139]
		49	Sanger sequencing	Exons 10 and 21 *	10%	---	[29]

* *PIK3CA* exon designation is done according to Ensembl Transcript ID: ENST00000263967.4, RefSeq: NM_006218.4. Exons 2, 3, 5, 6, 8, 10, 13, 14, 19 and 21 are often designated in the literature as coding exons 1, 2, 4, 5, 7, 9, 12, 13, 18, and 20, respectively. U/M, univariable or multivariable analysis; GBM, glioblastoma multiforme; PCR-SSCP, Polymerase Chain Reaction—Single Stranded Conformation Polymorphism; TCGA, The Cancer Genome Atlas.

**Table 2:** Current landscape of the main PI3K inhibitors in solid malignancies, including evidence from preclinical and clinical studies.

Inhibitor	Cancer Type	Cancer Subtype	Study Type	Treatment	Outcome	*PIK3CA* Status Dependent?	Reference
**Alpelisib** PI3Kα (orthosteric)	Breast	HR+ HER2-	Clinical-PhIII	+Fulvestrant	■ Favorable	♦ Yes	[158,159]
		HR+ HER2- PI3K altered	Clinical-PhII	Monotherapy	■ Favorable	N.E. (All-mut)	[160]
		Triple negative	Clinical-PhII	Monotherapy	■ No benefit	N.E.	
		---	Preclinical-in vitro	Monotherapy	■ Sensitive	♦ Yes	[161]
	Brain	GBM	Preclinical-in vivo	±PI3Kβ inhibitor (AZD6482)	■ Sensitive	N.E.	[162]
			Preclinical-in vitro	±mTOR inhibitor (OSI-027)	■ Sensitive	N.E. (WT-only)	[163]
	Gynecological	*PIK3CA*-mut	Clinical-obs	Monotherapy	■ Favorable	N.E. (All-mut)	[164]
		*PIK3CA*-mut cervical	Clinical	Monotherapy	■ Favorable	N.E. (All-mut)	[165]
		Cervical	Preclinical-in vitro	Monotherapy	■ Sensitive	♦ Yes	[166]
	Liver	HCC	Preclinical in vivo + in vitro	±mTOR inhibitor (MLN0128)	■ Sensitive	♦ Yes	[167]
	Head and neck	PI3K altered SCC	Clinical-PhII	Monotherapy	■ Favorable	♦ Yes	[168]
		SCC	Clinical-PhIb/II	+Cetuximab	■ No benefit	N.E.	[169]
			Preclinical-in vivo	Monotherapy	■ Sensitive	♦ Yes	[170]
			Preclinical-in vivo	+Cisplatin	■ Favorable	N.E. (All-mut)	[171]
	Lung	SCC	Preclinical-in vivo	±CDK4/6 inhibitors	■ Favorable	♦ Yes	[172]
**Buparlisib** Pan-PI3K (mainly PI3Kα)	Breast	HR+ HER2-	Clinical-PhIII	+Fulvestrant	■ Favorable	♦ Yes	[173]
			Clinical-PhII	+Tamoxifen	■ Favorable	♦ Yes	[174]
		Triple negative	Clinical-PhII	Monotherapy	■ Minimal benefit	N.E.	[175]
	Esophagus	SCC	Clinical-PhII	Monotherapy	■ Favorable	N.E.	[176]
	Brain	GBM	Clinical-PhII	Monotherapy	■ Minimal benefit	♦ No	[177]
			Clinical-PhIb/II	+Carboplatin or lomustine	■ Minimal benefit	N.E.	[178]
			Preclinical in vivo + in vitro	±PARP inhibitor (rucaparib)	■ Favorable	N.E. (WT-only)	[179]
			Preclinical-in vivo	Monotherapy	■ Minimal benefit	N.E.	[162]
			Preclinical-in vitro	±MEK inhibitor (selumetinib)	■ Sensitive	♦ No	[142]
	Head and neck	SCC	Clinical-PhII	±Cetuximab	■ Favorable	N.E. (WT-only)	[180]
			Clinical-PhII	+Paclitaxel	■ Favorable	♦ No	[181,182]
	Lung	SCC	Preclinical-in vivo	±CDK4/6 inhibitors	■ Favorable	♦ Yes	[172]
**Inavolisib** PI3Kα (orthosteric)	Breast	HR+ HER2- *PIK3CA*-mut	Clinical-PhIII	+Palbociclib-Fulvestrant	■ Favorable	N.E. (All-mut)	[183]
			Clinical-PhIb/II	+Letrozole or Fulvestrant	■ Favorable	N.E. (All-mut)	[184]
		---	Preclinical in vivo + in vitro	±Palbociclib and/or fulvestrant	■ Favorable	♦ Yes	[185]
			Preclinical-in vitro	Monotherapy	■ Sensitive	♦ Yes	[161]
**Paxalisib** Pan-PI3K/ mTOR	Brain	GBM	Clinical-PhII	Monotherapy	■ Favorable	N.E.	[186]
			Preclinical in vivo + in vitro	±EGFR inhibitor (AZD-9291)	■ Favorable	N.E. (WT-only)	[187]
			Preclinical-in vivo	Monotherapy	■ Sensitive	N.E.	[188,189]
**Pictilisib** PI3Kα/δ	Colon	---	Preclinical-in vitro	Monotherapy	■ Sensitive	♦ No	[185]
	Brain	GBM	Preclinical-in vitro	±Temozolomide	■ Sensitive	N.E.	[190]
	Head and neck	SCC	Preclinical-in vitro	Monotherapy	■ Sensitive	♦ No	[191,192]
**RLY-2608** PI3Kα (allosteric)	Breast	HR+ HER2- *PIK3CA*-mut	Clinical-case	+Fulvestrant	■ Favorable	N.E. (All-mut)	[193]
		---	Preclinical in vivo + in vitro	±Fulvestrant	■ Favorable	♦ Yes	
**Taselisib** PI3Kα/δ/γ	Breast	HR+ HER2-	Clinical-PhIII	+Fulvestrant	■ Minimal benefit	♦ No	[194]
			Clinical-PhII	+Fulvestrant	■ Favorable	♦ Yes	[195]
			Clinical-PhIb	+HER2 inhibitors	■ Favorable	♦ No	[196]
		---	Preclinical-in vivo	Monotherapy	■ Sensitive	N.E. (All-mut)	[185]
	Colon	---	Preclinical-in vitro	Monotherapy	■ Sensitive	♦ Yes	
	Head and neck	SCC	Preclinical in vivo + in vitro	Monotherapy	■ Favorable	♦ Yes	[191]
	Lung	SCC	Clinical-PhII	Monotherapy	■ No benefit	N.E. (All-mut)	[197]
**Voxtalisib** Pan-PI3K/ mTOR	Breast	HR+ HER2-	Clinical-PhI/II	+Letrozole	■ Minimal benefit	♦ No	[198]
	Brain	GBM	Clinical-PhI	+Temozolomide	■ Favorable	N.E.	[199]
			Preclinical in vivo + in vitro	±Temozolomide	■ Favorable	N.E. (WT-only)	[200]
		Low-grade glioma	Preclinical in vivo + in vitro	Monotherapy	■ Favorable	N.E. (WT-only)	[201]
	Gynecological	Ovarian	Clinical-PhII	+MEK inhibitor (pimasertib)	■ Minimal benefit	N.E.	[202]
		Endometrial	Preclinical-in vitro	±MEK inhibitor (pimasertib)	■ Sensitive	♦ No	[203]
	Prostate	---	Preclinical-in vitro	Monotherapy	■ Sensitive	N.E.	[204]

Legend: ■, Evidence supports added therapeutic benefit; ■, No evidence of added therapeutic benefit; ♦ Yes, response is dependent on *PIK3CA* mutational status; ♦ No, response is not dependent on *PIK3CA* mutational status; N.E., not evaluated (All-mut, all patients/models are *PIK3CA* mutated; WT-only, all patients/models are *PIK3CA* wildtype); GBM, glioblastoma multiforme; HCC, hepatocellular carcinoma; HER2, human epidermal growth factor receptor 2; HR, hormone receptor; obs, observational; SCC, squamous cell carcinoma.

## Abbreviations

The following abbreviations are used in this manuscript:

4E-BP1	Eukaryotic Translation Initiation Factor 4E-Binding Protein
ABD	Adaptor-Binding Domain
Akt	Protein Kinase B
ALT	Alanine Aminotransferase
AMPK	5′ Adenosine Monophosphate-Activated Protein Kinase
AST	Aspartate Aminotransferase
ATRX	Alpha Thalassemia/Mental Retardation Syndrome X-linked
BC	Breast Cancer
CDKN2A/B	Cyclin-Dependent Kinase Inhibitor 2A/B
CLL	Chronic Lymphocytic Leukemia
EGFR	Epidermal Growth Factor Receptor
eIF4E	Eukaryotic Translation Initiation Factor 4E
ERK	Extracellular Signal-Regulated Kinase
FDA	U.S. Food and Drug Administration
FKBP12	FK506 Binding Protein-12
FL	Follicular Lymphoma
FOXO	Forkhead Box O
GBM	Glioblastoma Multiforme
GFAP	Glial Fibrillary Acidic Protein
GPCR	G-Protein Coupled Receptors
GSK3α/β	Glycogen Synthase Kinase 3 α/β
HER2/3	Human Epidermal Growth Factor Receptor 2/3
HR	Hormone Receptor
IDH	Isocitrate Dehydrogenase
LSSC	Lung Squamous Cell Carcinoma
MAPK	Mitogen-Activated Protein Kinase
MGMT	O-6-Methylguanine-DNA Methyltransferase
mTOR	Mechanistic Target Of Rapamycin
mTORC1/2	Mechanistic Target Of Rapamycin Complex 1/2
NGS	Next-Generation Sequencing
OLIG2	Oligodendrocyte Lineage Transcription Factor 2
PCR-SSCP	Polymerase Chain Reaction—Single Stranded Conformation Polymorphism
PDK1	Phosphatidylinositol-Dependent Kinase 1
PFS	Progression-Free Survival
PI	Phosphatidylinositol
PI3K	Phosphatidylinositol-4,5-Bisphosphate 3-Kinase
PI3P	Phosphatidylinositol-3-Phosphate
PIK3CA	Phosphatidylinositol-4,5-Bisphosphate 3-Kinase Catalytic Subunit Alpha
PIP_2_	Phosphatidylinositol-4,5-Bisphosphate
PIP_3_	Phosphatidylinositol-3,4,5-Trisphosphate
PTEN	Phosphatase and Tensin Homologue
RBD	Ras-Binding Domain
RTK	Receptor Tyrosine Kinases
S6K	Ribosomal Protein S6 Kinase
SCC	Squamous Cell Carcinoma
SLL	Small Lymphocytic Lymphoma
TCGA	The Cancer Genome Atlas
TERT	Telomerase Reverse Transcriptase
TGFβ	Transforming Growth Factor β
TSC1/2	Tuberous Sclerosis Complex 1/2
VUS	Variants of Uncertain Significance
WHO	World Health Organization

## References

[1] Schaff L.R., Mellinghoff I.K. (2023). Glioblastoma and Other Primary Brain Malignancies in Adults: A Review. JAMA.

[2] Price M., Ballard C., Benedetti J., Neff C., Cioffi G., Waite K.A., Kruchko C., Barnholtz-Sloan J.S., Ostrom Q.T. (2024). CBTRUS Statistical Report: Primary Brain and Other Central Nervous System Tumors Diagnosed in the United States in 2017–2021. Neuro Oncol..

[3] Llaguno S.A., Chen J., Kwon C.-H., Jackson E.L., Li Y., Burns D.K., Alvarez-Buylla A., Parada L.F. (2009). Malignant Astrocytomas Originate from Neural Stem/Progenitor Cells in a Somatic Tumor Suppressor Mouse Model. Cancer Cell.

[4] Radke J., Bortolussi G., Pagenstecher A. (2013). Akt and C-Myc Induce Stem-Cell Markers in Mature Primary P53^−/−^ Astrocytes and Render These Cells Gliomagenic in the Brain of Immunocompetent Mice. PLoS ONE.

[5] Lindberg N., Kastemar M., Olofsson T., Smits A., Uhrbom L. (2009). Oligodendrocyte Progenitor Cells Can Act as Cell of Origin for Experimental Glioma. Oncogene.

[6] Velásquez C., Mansouri S., Mora C., Nassiri F., Suppiah S., Martino J., Zadeh G., Fernández-Luna J.L. (2019). Molecular and Clinical Insights into the Invasive Capacity of Glioblastoma Cells. J. Oncol..

[7] Rouse C., Gittleman H., Ostrom Q.T., Kruchko C., Barnholtz-Sloan J.S. (2016). Years of Potential Life Lost for Brain and CNS Tumors Relative to Other Cancers in Adults in the United States, 2010. Neuro Oncol..

[8] Stupp R., Mason W.P., van den Bent M.J., Weller M., Fisher B., Taphoorn M.J.B., Belanger K., Brandes A.A., Marosi C., Bogdahn U. (2005). Radiotherapy plus Concomitant and Adjuvant Temozolomide for Glioblastoma. N. Engl. J. Med..

[9] Kalokhe G., Grimm S.A., Chandler J.P., Helenowski I., Rademaker A., Raizer J.J. (2012). Metastatic Glioblastoma: Case Presentations and a Review of the Literature. J. Neuro-Oncol..

[10] Bao S., Wu Q., McLendon R.E., Hao Y., Shi Q., Hjelmeland A.B., Dewhirst M.W., Bigner D.D., Rich J.N. (2006). Glioma Stem Cells Promote Radioresistance by Preferential Activation of the DNA Damage Response. Nature.

[11] Hombach-Klonisch S., Mehrpour M., Shojaei S., Harlos C., Pitz M., Hamai A., Siemianowicz K., Likus W., Wiechec E., Toyota B.D. (2018). Glioblastoma and Chemoresistance to Alkylating Agents: Involvement of Apoptosis, Autophagy, and Unfolded Protein Response. Pharmacol. Ther..

[12] Louis D.N., Ohgaki H., Wiestler O.D., Cavenee W.K., Burger P.C., Jouvet A., Scheithauer B.W., Kleihues P. (2007). The 2007 WHO Classification of Tumours of the Central Nervous System. Acta Neuropathol..

[13] Louis D.N., Perry A., Reifenberger G., von Deimling A., Figarella D., Webster B., Hiroko K.C., Wiestler O.D., Kleihues P., Ellison D.W. (2016). The 2016 World Health Organization Classification of Tumors of the Central Nervous System: A Summary. Acta Neuropathol..

[14] Brat D.J., Prayson R.A., Ryken T.C., Olson J.J. (2008). Diagnosis of Malignant Glioma: Role of Neuropathology. J. Neurooncol.

[15] Reifenberger G., Wirsching H.-G., Knobbe-Thomsen C.B., Weller M. (2017). Advances in the Molecular Genetics of Gliomas—Implications for Classification and Therapy. Nat. Rev. Clin. Oncol..

[16] Brito C., Azevedo A., Esteves S., Marques A.R., Martins C., Costa I., Mafra M., Marques J.M.B., Roque L., Pojo M. (2019). Clinical Insights Gained by Refining the 2016 WHO Classification of Diffuse Gliomas with: EGFR Amplification, TERT Mutations, PTEN Deletion and MGMT Methylation. BMC Cancer.

[17] Ceccarelli M., Barthel F.P., Malta T.M., Sabedot T.S., Salama S.R., Murray B.A., Morozova O., Newton Y., Radenbaugh A., Pagnotta S.M. (2016). Molecular Profiling Reveals Biologically Discrete Subsets and Pathways of Progression in Diffuse Glioma. Cell.

[18] Brennan C.W., Verhaak R.G.W., McKenna A., Campos B., Noushmehr H., Salama S.R., Zheng S., Chakravarty D., Sanborn J.Z., Berman S.H. (2013). The Somatic Genomic Landscape of Glioblastoma. Cell.

[19] Louis D.N., Perry A., Wesseling P., Brat D.J., Cree I.A., Figarella-Branger D., Hawkins C., Ng H.K., Pfister S.M., Reifenberger G. (2021). The 2021 WHO Classification of Tumors of the Central Nervous System: A Summary. Neuro Oncol..

[20] Beiko J., Suki D., Hess K.R., Fox B.D., Cheung V., Cabral M., Shonka N., Gilbert M.R., Sawaya R., Prabhu S.S. (2014). IDH1 Mutant Malignant Astrocytomas Are More Amenable to Surgical Resection and Have a Survival Benefit Associated with Maximal Surgical Resection. Neuro Oncol..

[21] Cairncross J.G., Wang M., Jenkins R.B., Shaw E.G., Giannini C., Brachman D.G., Buckner J.C., Fink K.L., Souhami L., Laperriere N.J. (2014). Benefit From Procarbazine, Lomustine and Vincristine in Oligodendroglial Tumors Is Associated With Mutation of IDH. J. Clinl Oncol..

[22] Weller M., van den Bent M., Preusser M., Le Rhun E., Tonn J.C., Minniti G., Bendszus M., Balana C., Chinot O., Dirven L. (2021). EANO Guidelines on the Diagnosis and Treatment of Diffuse Gliomas of Adulthood. Nat. Rev. Clin. Oncol..

[23] Jenkins R.B., Blair H., Ballman K.V., Giannini C., Arusell R.M., Law M., Flynn H., Passe S., Felten S., Brown P.D. (2006). A t(1;19)(Q10;P10) Mediates the Combined Deletions of 1p and 19q and Predicts a Better Prognosis of Patients with Oligodendroglioma. Cancer Res..

[24] Weller M., Weber R.G., Willscher E., Riehmer V., Hentschel B., Kreuz M., Felsberg J., Beyer U., Loffler-Wirth H., Kaulich K. (2015). Molecular Classification of Diffuse Cerebral WHO Grade II/III Gliomas Using Genome- and Transcriptome-Wide Profiling Improves Stratification of Prognostically Distinct Patient Groups. Acta Neuropathol..

[25] Eckel-Passow J.E., Lachance D.H., Molinaro A.M., Walsh K.M., Decker P.A., Sicotte H., Pekmezci M., Rice T., Kosel M.L., Smirnov I.V. (2015). Glioma Groups Based on 1p/19q, IDH, and TERT Promoter Mutations in Tumors. N. Engl. J. Med..

[26] Lee Y., Koh J., Kim S.-I., Won J.K., Park C.-K., Choi S.H., Park S.-H. (2017). The Frequency and Prognostic Effect of TERT Promoter Mutation in Diffuse Gliomas. Acta Neuropathol. Commun..

[27] Lee J.K., Wang J., Sa J.K., Ladewig E., Lee H.-O., Lee I.-H., Kang H.J., Rosenbloom D.S., Camara P.G., Liu Z. (2017). Spatiotemporal Genomic Architecture Informs Precision Oncology in Glioblastoma. Nat. Genet..

[28] Tanaka S., Batchelor T.T., Iafrate A.J., Dias-Santagata D., Borger D.R., Ellisen L.W., Yang D., Louis D.N., Cahill D.P., Chi A.S. (2019). PIK3CA Activating Mutations Are Associated with More Disseminated Disease at Presentation and Earlier Recurrence in Glioblastoma. Acta Neuropathol. Commun..

[29] Brito C., Tomás A., Azevedo A., Esteves S., Mafra M., Roque L., Pojo M. (2022). PIK3CA Mutations in Diffuse Gliomas: An Update on Molecular Stratification, Prognosis, Recurrence, and Aggressiveness. Clin. Med. Insights Oncol..

[30] Lai K., Killingsworth M.C., Lee C.S. (2015). Gene of the Month: PIK3CA. J. Clin. Pathol..

[31] Engelman J.A., Luo J., Cantley L.C. (2006). The Evolution of Phosphatidylinositol 3-Kinases as Regulators of Growth and Metabolism. Nat. Rev. Genet..

[32] Gaidarov I., Smith M.E.K., Domin J., Keen J.H. (2001). The Class II Phosphoinositide 3-Kinase C2α Is Activated by Clathrin and Regulates Clathrin-Mediated Membrane Trafficking. Mol. Cell.

[33] Yoshioka K., Yoshida K., Cui H., Wakayama T., Takuwa N., Okamoto Y., Du W., Qi X., Asanuma K., Sugihara K. (2012). Endothelial PI3K-C2α, a Class II PI3K, Has an Essential Role in Angiogenesis and Vascular Barrier Function. Nat. Med..

[34] Wurmser A.E., Emr S.D. (2002). Novel PtdIns(3)P-Binding Protein Etf1 Functions as an Effector of the Vps34 PtdIns 3-Kinase in Autophagy. J. Cell Biol..

[35] Nobukuni T., Joaquin M., Roccio M., Dann S.G., Kim S.Y., Gulati P., Byfield M.P., Backer J.M., Natt F., Bos J.L. (2005). Amino Acids Mediate MTOR/Raptor Signaling through Activation of Class 3 Phosphatidylinositol 3OH-Kinase. Proc. Natl. Acad. Sci. USA.

[36] Stoyanov B., Volinia S., Hanck T., Rubio I., Loubtchenkov M., Malek D., Stoyanova S., Vanhaesebroeck B., Dhand R., Nürnberg B. (1995). Cloning and Characterization of a G Protein-Activated Human Phosphoinositide-3 Kinase. Science.

[37] Stephens L.R., Eguinoa A., Erdjument-Bromage H., Lui M., Cooke F., Coadwell J., Smrcka A.S., Thelen M., Cadwallader K., Tempst P. (1997). The G Beta Gamma Sensitivity of a PI3K Is Dependent upon a Tightly Associated Adaptor, P101. Cell.

[38] Willis O., Choucair K., Alloghbi A., Stanbery L., Mowat R., Brunicardi F.C., Dworkin L., Nemunaitis J. (2020). PIK3CA Gene Aberrancy and Role in Targeted Therapy of Solid Malignancies. Cancer Gene Ther..

[39] Okkenhaug K., Bilancio A., Priddle H., Sancho S., Peskett E., Pearce W., Meek S.E., Salpekar A., Waterfield M.D., Smith A.J.H. (2002). Impaired B and T Cell Antigen Receptor Signaling in P110delta PI 3-Kinase Mutant Mice. Science.

[40] Burke J.E. (2018). Structural Basis for Regulation of Phosphoinositide Kinases and Their Involvement in Human Disease. Mol. Cell.

[41] Yu J., Zhang Y., McIlroy J., Rordorf-Nikolic T., Orr G.A., Backer J.M. (1998). Regulation of the P85/P110 Phosphatidylinositol 3′-Kinase: Stabilization and Inhibition of the P110α Catalytic Subunit by the P85 Regulatory Subunit. Mol. Cell Biol..

[42] Klippel A., Kavanaugh W.M., Pot D., Williams L.T. (1997). A Specific Product of Phosphatidylinositol 3-Kinase Directly Activates the Protein Kinase Akt through Its Pleckstrin Homology Domain. Mol. Cell Biol..

[43] Iida M., Harari P.M., Wheeler D.L., Toulany M. (2020). Targeting AKT/PKB to Improve Treatment Outcomes for Solid Tumors. Mutat. Res..

[44] Alessi D.R., James S.R., Downes C.P., Holmes A.B., Gaffney P.R.J., Reese C.B., Cohen P. (1997). Characterization of a 3-Phosphoinositide-Dependent Protein Kinase Which Phosphorylates and Activates Protein Kinase Bα. Curr. Biol..

[45] Sarbassov D.D., Guertin D.A., Ali S.M., Sabatini D.M. (2005). Phosphorylation and Regulation of Akt/PKB by the Rictor-MTOR Complex. Science.

[46] Frame S., Cohen P., Biondi R.M. (2001). A Common Phosphate Binding Site Explains the Unique Substrate Specificity of GSK3 and Its Inactivation by Phosphorylation. Mol. Cell.

[47] Brunet A., Bonni A., Zigmond M.J., Lin M.Z., Juo P., Hu L.S., Anderson M.J., Arden K.C., Blenis J., Greenberg M.E. (1999). Akt Promotes Cell Survival by Phosphorylating and Inhibiting a Forkhead Transcription Factor. Cell.

[48] Potter C.J., Pedraza L.G., Xu T. (2002). Akt Regulates Growth by Directly Phosphorylating Tsc2. Nat. Cell Biol..

[49] Inoki K., Li Y., Zhu T., Wu J., Guan K.-L. (2002). TSC2 Is Phosphorylated and Inhibited by Akt and Suppresses MTOR Signalling. Nat. Cell Biol..

[50] Maehama T., Dixon J.E. (1998). The Tumor Suppressor, PTEN/MMAC1, Dephosphorylates the Lipid Second Messenger, Phosphatidylinositol 3,4,5-Trisphosphate. J. Biol. Chem..

[51] Castellano E., Molina-Arcas M., Krygowska A.A., East P., Warne P., Nicol A., Downward J. (2016). RAS Signalling through PI3-Kinase Controls Cell Migration via Modulation of Reelin Expression. Nat. Commun..

[52] Zimmermann S., Moelling K. (1999). Phosphorylation and Regulation of Raf by Akt (Protein Kinase B). Science.

[53] Hawley S.A., Ross F.A., Gowans G.J., Tibarewal P., Leslie N.R., Hardie D.G. (2014). Phosphorylation by Akt within the ST Loop of AMPK-A1 down-Regulates Its Activation in Tumour Cells. Biochem. J..

[54] Fang D., Hawke D., Zheng Y., Xia Y., Meisenhelder J., Nika H., Mills G.B., Kobayashi R., Hunter T., Lu Z. (2007). Phosphorylation of β-Catenin by AKT Promotes β-Catenin Transcriptional Activity. J. Biol. Chem..

[55] Hamidi A., Song J., Thakur N., Itoh S., Marcusson A., Bergh A., Heldin C.-H., Landström M. (2017). TGF-β Promotes PI3K-AKT Signaling and Prostate Cancer Cell Migration through the TRAF6-Mediated Ubiquitylation of P85α. Sci. Signal.

[56] Millis S.Z., Jardim D.L., Albacker L., Ross J.S., Miller V.A., Ali S.M., Kurzrock R. (2019). Phosphatidylinositol 3-Kinase Pathway Genomic Alterations in 60,991 Diverse Solid Tumors Informs Targeted Therapy Opportunities. Cancer.

[57] Kandoth C., McLellan M.D., Vandin F., Ye K., Niu B., Lu C., Xie M., Zhang Q., McMichael J.F., Wyczalkowski M.A. (2013). Mutational Landscape and Significance across 12 Major Cancer Types. Nature.

[58] Thorpe L.M., Yuzugullu H., Zhao J.J. (2015). PI3K in Cancer: Divergent Roles of Isoforms, Modes of Activation and Therapeutic Targeting. Nat. Rev. Cancer.

[59] Xiao W., Zhang G., Chen B., Chen X., Wen L., Lai J., Li X., Li M., Liu H., Liu J. (2021). Mutational Landscape of PI3K-AKT-MTOR Pathway in Breast Cancer: Implications for Targeted Therapeutics. J. Cancer.

[60] Samuels Y., Wang Z., Bardelli A., Silliman N., Ptak J., Szabo S., Yan H., Gazdar A., Powell S.M., Riggins G.J. (2004). High Frequency of Mutations of the PIK3CA Gene in Human Cancers. Science.

[61] Kang S., Bader A.G., Vogt P.K. (2005). Phosphatidylinositol 3-Kinase Mutations Identified in Human Cancer Are Oncogenic. Proc. Natl. Acad. Sci. USA.

[62] Samuels Y., Diaz L.A., Schmidt-Kittler O., Cummins J.M., DeLong L., Cheong I., Rago C., Huso D.L., Lengauer C., Kinzler K.W. (2005). Mutant PIK3CA Promotes Cell Growth and Invasion of Human Cancer Cells. Cancer Cell.

[63] Bader A.G., Kang S., Vogt P.K. (2006). Cancer-Specific Mutations in PIK3CA Are Oncogenic in Vivo. Proc. Natl. Acad. Sci. USA.

[64] Isakoff S.J., Engelman J.A., Irie H.Y., Luo J., Brachmann S.M., Pearline R.V., Cantley L.C., Brugge J.S. (2005). Breast Cancer-Associated PIK3CA Mutations Are Oncogenic in Mammary Epithelial Cells. Cancer Res..

[65] Zhao L., Vogt P.K. (2010). Hot-Spot Mutations in P110α of Phosphatidylinositol 3-Kinase (PI3K): Differential Interactions with the Regulatory Subunit P85 and with RAS. Cell Cycle.

[66] Gkeka P., Evangelidis T., Pavlaki M., Lazani V., Christoforidis S., Agianian B., Cournia Z. (2014). Investigating the Structure and Dynamics of the PIK3CA Wild-Type and H1047R Oncogenic Mutant. PLoS Comput. Biol..

[67] Leontiadou H., Galdadas I., Athanasiou C., Cournia Z. (2018). Insights into the Mechanism of the PIK3CA E545K Activating Mutation Using MD Simulations. Sci. Rep..

[68] Miled N., Yan Y., Hon W.C., Perisic O., Zvelebil M., Inbar Y., Schneidman-Duhovny D., Wolfson H.J., Backer J.M., Williams R.L. (2007). Mechanism of Two Classes of Cancer Mutations in the Phosphoinositide 3-Kinase Catalytic Subunit. Science.

[69] Jenkins M.L., Ranga-Prasad H., Parson M.A.H., Harris N.J., Rathinaswamy M.K., Burke J.E. (2023). Oncogenic Mutations of PIK3CA Lead to Increased Membrane Recruitment Driven by Reorientation of the ABD, P85 and C-Terminus. Nat. Commun..

[70] Gymnopoulos M., Elsliger M.-A., Vogt P.K. (2007). Rare Cancer-Specific Mutations in PIK3CA Show Gain of Function. Proc. Natl. Acad. Sci. USA.

[71] Gabelli S.B., Huang C.-H., Mandelker D., Schmidt-Kittler O., Vogelstein B., Amzel L.M. (2010). Structural Effects of Oncogenic PI3Ka Mutations. Curr. Top. Microbiol. Immunol..

[72] Yuan W., Goldstein L.D., Durinck S., Chen Y.-J., Nguyen T.T., Kljavin N.M., Sokol E.S., Stawiski E.W., Haley B., Ziai J. (2019). S100a4 Upregulation in Pik3caH1047R;Trp53R270H;MMTV-Cre-Driven Mammary Tumors Promotes Metastasis. Breast Cancer Res..

[73] The Cancer Genome Atlas Network (2012). Comprehensive Molecular Portraits of Human Breast Tumours. Nature.

[74] Mangone F.R., Bobrovnitchaia I.G., Salaorni S., Manuli E., Nagai M.A. (2012). PIK3CA Exon 20 Mutations Are Associated with Poor Prognosis in Breast Cancer Patients. Clinics.

[75] Kalinsky K., Jacks L.M., Heguy A., Patil S., Drobnjak M., Bhanot U.K., Hedvat C.V., Traina T.A., Solit D., Gerald W. (2009). PIK3CA Mutation Associates with Improved Outcome in Breast Cancer. Clin. Cancer Res..

[76] Li S.Y., Rong M., Grieu F., Iacopetta B. (2006). PIK3CA Mutations in Breast Cancer Are Associated with Poor Outcome. Breast Cancer Res. Treat..

[77] Loi S., Haibe-Kains B., Majjaj S., Lallemand F., Durbecq V., Larsimont D., Gonzalez-Angulo A.M., Pusztai L., Symmans W.F., Bardelli A. (2010). PIK3CA Mutations Associated with Gene Signature of Low MTORC1 Signaling and Better Outcomes in Estrogen Receptor-Positive Breast Cancer. Proc. Natl. Acad. Sci. USA.

[78] Deng L., Zhu X., Sun Y., Wang J., Zhong X., Li J., Hu M., Zheng H. (2019). Prevalence and Prognostic Role of PIK3CA/AKT1 Mutations in Chinese Breast Cancer Patients. Cancer Res. Treat..

[79] Stemke-Hale K., Gonzalez-Angulo A.M., Lluch A., Neve R.M., Kuo W.-L., Davies M., Carey M., Hu Z., Guan Y., Sahin A. (2008). An Integrative Genomic and Proteomic Analysis of PIK3CA, PTEN, and AKT Mutations in Breast Cancer. Cancer Res..

[80] Martínez-Saéz O., Chic N., Pascual T., Adamo B., Vidal M., González-Farré B., Sanfeliu E., Schettini F., Conte B., Brasó-Maristany F. (2020). Frequency and Spectrum of PIK3CA Somatic Mutations in Breast Cancer. Breast Cancer Res..

[81] Reinhardt K., Stückrath K., Hartung C., Kaufhold S., Uleer C., Hanf V., Lantzsch T., Peschel S., John J., Pöhler M. (2022). PIK3CA-Mutations in Breast Cancer. Breast Cancer Res. Treat..

[82] Loibl S., von Minckwitz G., Schneeweiss A., Paepke S., Lehmann A., Rezai M., Zahm D.M., Sinn P., Khandan F., Eidtmann H. (2014). PIK3CA Mutations Are Associated with Lower Rates of Pathologic Complete Response to Anti-Human Epidermal Growth Factor Receptor 2 (HER2) Therapy in Primary HER2-Overexpressing Breast Cancer. J. Clin. Oncol..

[83] Kim J.-Y., Lee E., Park K., Park W.-Y., Jung H.H., Ahn J.S., Im Y.-H., Park Y.H. (2017). Clinical Implications of Genomic Profiles in Metastatic Breast Cancer with a Focus on TP53 and PIK3CA, the Most Frequently Mutated Genes. Oncotarget.

[84] Razavi P., Chang M.T., Xu G., Bandlamudi C., Ross D.S., Vasan N., Cai Y., Bielski C.M., Donoghue M.T.A., Jonsson P. (2018). The Genomic Landscape of Endocrine-Resistant Advanced Breast Cancers. Cancer Cell.

[85] Sobhani N., Roviello G., Corona S.P., Scaltriti M., Ianza A., Bortul M., Zanconati F., Generali D. (2018). The Prognostic Value of PI3K Mutational Status in Breast Cancer: A Meta-Analysis. J. Cell Biochem..

[86] Hart J.R., Zhang Y., Liao L., Ueno L., Du L., Jonkers M., Yates J.R., Vogt P.K. (2015). The Butterfly Effect in Cancer: A Single Base Mutation Can Remodel the Cell. Proc. Natl. Acad. Sci. USA.

[87] Meyer D.S., Koren S., Leroy C., Brinkhaus H., Müller U., Klebba I., Müller M., Cardiff R.D., Bentires-Alj M. (2013). Expression of PIK3CA Mutant E545K in the Mammary Gland Induces Heterogeneous Tumors but Is Less Potent than Mutant H1047R. Oncogenesis.

[88] Guo S., Loibl S., von Minckwitz G., Darb-Esfahani S., Lederer B., Denkert C. (2020). PIK3CA H1047R Mutation Associated with a Lower Pathological Complete Response Rate in Triple-Negative Breast Cancer Patients Treated with Anthracycline-Taxane–Based Neoadjuvant Chemotherapy. Cancer Res. Treat..

[89] Mosele F., Stefanovska B., Lusque A., Dien A.T., Garberis I., Droin N., Le Tourneau L., Sablin M., Lacroix L., Enrico D. (2020). Outcome and Molecular Landscape of Patients with PIK3CA-Mutated Metastatic Breast Cancer. Ann. Oncol..

[90] Kim J.W., Lim A.R., You J.Y., Lee J.H., Song S.E., Lee N.K., Jung S.P., Cho K.R., Kim C.Y., Park K.H. (2023). PIK3CA Mutation Is Associated with Poor Response to HER2-Targeted Therapy in Breast Cancer Patients. Cancer Res. Treat..

[91] Cizkova M., Dujaric M.-E., Lehmann-Che J., Scott V., Tembo O., Asselain B., Pierga J.-Y., Marty M., De Cremoux P., Spyratos F. (2013). Outcome Impact of PIK3CA Mutations in HER2-Positive Breast Cancer Patients Treated with Trastuzumab. Br. J. Cancer.

[92] Berns K., Horlings H.M., Hennessy B.T., Madiredjo M., Hijmans E.M., Beelen K., Linn S.C., Gonzalez-Angulo A.M., Stemke-Hale K., Hauptmann M. (2007). A Functional Genetic Approach Identifies the PI3K Pathway as a Major Determinant of Trastuzumab Resistance in Breast Cancer. Cancer Cell.

[93] Chiu J.W., Krzyzanowska M.K., Serra S., Knox J.J., Dhani N.C., Mackay H., Hedley D., Moore M., Liu G., Burkes R.L. (2018). Molecular Profiling of Patients with Advanced Colorectal Cancer: Princess Margaret Cancer Centre Experience. Clin. Color. Cancer.

[94] Stec R., Semeniuk-Wojtaś A., Charkiewicz R., Bodnar L., Korniluk J., Smoter M., Chyczewski L., Nikliński J., Szczylik C. (2015). Mutation of the PIK3CA Gene as a Prognostic Factor in Patients with Colorectal Cancer. Oncol. Lett..

[95] Ogino S., Liao X., Imamura Y., Yamauchi M., McCleary N.J., Ng K., Niedzwiecki D., Saltz L.B., Mayer R.J., Whittom R. (2013). Predictive and Prognostic Analysis of PIK3CA Mutation in Stage III Colon Cancer Intergroup Trial. J. Natl. Cancer Inst..

[96] Day F.L., Jorissen R.N., Lipton L., Mouradov D., Sakthianandeswaren A., Christie M., Li S., Tsui C., Tie J., Desai J. (2013). PIK3CA and PTEN Gene and Exon Mutation-Specific Clinicopathologic and Molecular Associations in Colorectal Cancer. Clin. Cancer Res..

[97] Lee C.S., Song I.H., Lee A., Kang J., Lee Y.S., Lee I.K., Song Y.S., Lee S.H. (2021). Enhancing the Landscape of Colorectal Cancer Using Targeted Deep Sequencing. Sci. Rep..

[98] Fu X., Lin H., Fan X., Zhu Y., Wang C., Chen Z., Tan X., Huang J., Cai Y., Huang Y. (2021). The Spectrum, Tendency and Predictive Value of PIK3CA Mutation in Chinese Colorectal Cancer Patients. Front. Oncol..

[99] Wang Q., Shi Y., Zhou K., Wang L., Yan Z., Liu Y., Xu L., Zhao S., Chu H., Shi T. (2018). PIK3CA Mutations Confer Resistance to First-Line Chemotherapy in Colorectal Cancer. Cell Death Dis..

[100] Ogino S., Nosho K., Kirkner G.J., Shima K., Irahara N., Kure S., Chan A.T., Engelman J.A., Kraft P., Cantley L.C. (2009). PIK3CA Mutation Is Associated with Poor Prognosis among Patients with Curatively Resected Colon Cancer. J. Clin. Oncol..

[101] Sartore-Bianchi A., Martini M., Molinari F., Veronese S., Nichelatti M., Artale S., Di Nicolantonio F., Saletti P., De Dosso S., Mazzucchelli L. (2009). PIK3CA Mutations in Colorectal Cancer Are Associated with Clinical Resistance to EGFR-Targeted Monoclonal Antibodies. Cancer Res..

[102] Ranjbar R., Mohammadpour S., Esfahani A.T., Namazian S., Yaghob-Taleghani M., Baghaei K., Tabatabaei S.A.M., Pasharavesh L., Nazemalhosseini-Mojarad E. (2019). Prevalence and Prognostic Role of PIK3CA E545K Mutation in Iranian Colorectal Cancer Patients. Gastroenterol. Hepatol. Bed Bench.

[103] Jin J., Shi Y., Zhang S., Yang S. (2020). PIK3CA Mutation and Clinicopathological Features of Colorectal Cancer: A Systematic Review and Meta-Analysis. Acta Oncol..

[104] Xu J.-M., Wang Y., Wang Y.-L., Wang Y., Liu T., Ni M., Li M.-S., Lin L., Ge F.-J., Gong C. (2017). PIK3CA Mutations Contribute to Acquired Cetuximab Resistance in Patients with Metastatic Colorectal Cancer. Clin. Cancer Res..

[105] Polom K., Marrelli D., Roviello G., Pascale V., Voglino C., Vindigni C., Generali D., Roviello F. (2018). PIK3CA Mutation in Gastric Cancer and the Role of Microsatellite Instability Status in Mutations of Exons 9 and 20 of the PIK3CA Gene. Adv. Clin. Exp. Med..

[106] Fang W.-L., Huang K.-H., Lan Y.-T., Lin C.-H., Chang S.-C., Chen M.-H., Chao Y., Lin W.-C., Lo S.-S., Li A.-F.Y. (2016). Mutations in PI3K/AKT Pathway Genes and Amplifications of PIK3CA Are Associated with Patterns of Recurrence in Gastric Cancers. Oncotarget.

[107] Takahashi N., Yamada Y., Taniguchi H., Fukahori M., Sasaki Y., Shoji H., Honma Y., Iwasa S., Takashima A., Kato K. (2014). Clinicopathological Features and Prognostic Roles of KRAS, BRAF, PIK3CA and NRAS Mutations in Advanced Gastric Cancer. BMC Res. Notes.

[108] Barbi S., Cataldo I., De Manzoni G., Bersani S., Lamba S., Mattuzzi S., Bardelli A., Scarpa A. (2010). The Analysis of PIK3CA Mutations in Gastric Carcinoma and Metanalysis of Literature Suggest That Exon-Selectivity Is a Signature of Cancer Type. J. Exp. Clin. Cancer Res..

[109] Kim J.-W., Lee H.S., Nam K.H., Ahn S., Kim J.W., Ahn S.-H., Park D.J., Kim H.-H., Lee K.-W. (2017). PIK3CA Mutations Are Associated with Increased Tumor Aggressiveness and Akt Activation in Gastric Cancer. Oncotarget.

[110] Harada K., Baba Y., Shigaki H., Ishimoto T., Miyake K., Kosumi K., Tokunaga R., Izumi D., Ohuchi M., Nakamura K. (2016). Prognostic and Clinical Impact of PIK3CA Mutation in Gastric Cancer: Pyrosequencing Technology and Literature Review. BMC Cancer.

[111] The Cancer Genome Atlas Research Network (2014). Comprehensive Molecular Characterization of Gastric Adenocarcinoma. Nature.

[112] Velasco A., Bussaglia E., Pallares J., Dolcet X., Llobet D., Encinas M., Llecha N., Palacios J., Prat J., Matias-Guiu X. (2006). PIK3CA Gene Mutations in Endometrial Carcinoma: Correlation with PTEN and K-RAS Alterations. Hum. Pathol..

[113] Watanabe T., Nanamiya H., Kojima M., Nomura S., Furukawa S., Soeda S., Tanaka D., Isogai T., Imai J.-i., Watanabe S. (2021). Clinical Relevance of Oncogenic Driver Mutations Identified in Endometrial Carcinoma. Transl. Oncol..

[114] Oda K., Stokoe D., Taketani Y., McCormick F. (2005). High Frequency of Coexistent Mutations of PIK3CA and PTEN Genes in Endometrial Carcinoma. Cancer Res..

[115] Mjos S., Werner H.M.J., Birkeland E., Holst F., Berg A., Halle M.K., Tangen I.L., Kusonmano K., Mauland K.K., Oyan A.M. (2017). PIK3CA Exon9 Mutations Associate with Reduced Survival, and Are Highly Concordant between Matching Primary Tumors and Metastases in Endometrial Cancer. Sci. Rep..

[116] Nakagaki T., Tamura M., Kobashi K., Omori A., Koyama R., Idogawa M., Ogi K., Hiratsuka H., Tokino T., Sasaki Y. (2018). Targeted Next-Generation Sequencing of 50 Cancer-Related Genes in Japanese Patients with Oral Squamous Cell Carcinoma. Tumor Biol..

[117] The Cancer Genome Atlas Research Network (2015). Comprehensive Genomic Characterization of Head and Neck Squamous Cell Carcinomas. Nature.

[118] Morris L.G.T., Taylor B.S., Bivona T.G., Gong Y., Eng S., Brennan C.W., Kaufman A., Kastenhuber E.R., Banuchi V.E., Singh B. (2011). Genomic Dissection of the Epidermal Growth Factor Receptor (EGFR)/PI3K Pathway Reveals Frequent Deletion of the EGFR Phosphatase PTPRS in Head and Neck Cancers. Proc. Natl. Acad. Sci. USA.

[119] Agrawal N., Frederick M.J., Pickering C.R., Bettegowda C., Chang K., Li R.J., Fakhry C., Xie T.-X., Zhang J., Wang J. (2011). Exome Sequencing of Head and Neck Squamous Cell Carcinoma Reveals Inactivating Mutations in NOTCH1. Science.

[120] Lui V.W.Y., Hedberg M.L., Li H., Vangara B.S., Pendleton K., Zeng Y., Lu Y., Zhang Q., Du Y., Gilbert B.R. (2013). Frequent Mutation of the PI3K Pathway in Head and Neck Cancer Defines Predictive Biomarkers. Cancer Discov..

[121] Yokota T., Serizawa M., Hosokawa A., Kusafuka K., Mori K., Sugiyama T., Tsubosa Y., Koh Y. (2018). PIK3CA Mutation Is a Favorable Prognostic Factor in Esophageal Cancer: Molecular Profile by next-Generation Sequencing Using Surgically Resected Formalin-Fixed, Paraffin-Embedded Tissue. BMC Cancer.

[122] Hou J., Jiang D., Zhang J., Gavine P.R., Xu S., Liu Y., Xu C., Huang J., Tan Y., Wang H. (2014). Frequency, Characterization, and Prognostic Analysis of PIK3CA Gene Mutations in Chinese Esophageal Squamous Cell Carcinoma. Hum. Pathol..

[123] Shigaki H., Baba Y., Watanabe M., Murata A., Ishimoto T., Iwatsuki M., Iwagami S., Nosho K., Baba H. (2013). PIK3CA Mutation Is Associated with a Favorable Prognosis among Patients with Curatively Resected Esophageal Squamous Cell Carcinoma. Clin. Cancer Res..

[124] Zheng H., Wang Y., Tang C., Jones L., Ye H., Zhang G., Cao W., Li J., Liu L., Liu Z. (2016). TP53, PIK3CA, FBXW7 and KRAS Mutations in Esophageal Cancer Identified by Targeted Sequencing. Cancer Genom. Proteom..

[125] Sawada G., Niida A., Uchi R., Hirata H., Shimamura T., Suzuki Y., Shiraishi Y., Chiba K., Imoto S., Takahashi Y. (2016). Genomic Landscape of Esophageal Squamous Cell Carcinoma in a Japanese Population. Gastroenterology.

[126] Gao Y.-B., Chen Z.-L., Li J.-G., Hu X.-D., Shi X.-J., Sun Z.-M., Zhang F., Zhao Z.-R., Li Z.-T., Liu Z.-Y. (2014). Genetic Landscape of Esophageal Squamous Cell Carcinoma. Nat. Genet..

[127] Seo A.N., Kang B.W., Bae H.I., Kwon O.K., Park K.B., Lee S.S., Chung H.Y., Yu W., Jeon S.W., Kang H. (2019). Exon 9 Mutation of PIK3CA Associated with Poor Survival in Patients with Epstein-Barr Virus-Associated Gastric Cancer. Anticancer. Res..

[128] Bredin H.K., Krakstad C., Hoivik E.A. (2023). PIK3CA Mutations and Their Impact on Survival Outcomes of Patients with Endometrial Cancer: A Systematic Review and Meta-Analysis. PLoS ONE.

[129] Broderick D.K., Di C., Parrett T.J., Samuels Y.R., Cummins J.M., Mclendon R.E., Fults D.W., Velculescu V.E., Bigner D.D., Yan H. (2004). Mutations of PIK3CA in Anaplastic Oligodendrogliomas, High-Grade Astrocytomas, and Medulloblastomas. Cancer Res..

[130] Gallia G.L., Rand V., Siu I.-M., Eberhart C.G., James C.D., Marie S.K.N., Oba-Shinjo S.M., Carlotti C.G., Caballero O.L., Simpson A.J.G. (2006). PIK3CA Gene Mutations in Pediatric and Adult Glioblastoma Multiforme. Mol. Cancer Res..

[131] Verhaak R.G.W., Hoadley K.A., Purdom E., Wang V., Qi Y., Wilkerson M.D., Miller C.R., Ding L., Golub T., Mesirov J.P. (2010). Integrated Genomic Analysis Identifies Clinically Relevant Subtypes of Glioblastoma Characterized by Abnormalities in PDGFRA, IDH1, EGFR and NF1. Cancer Cell.

[132] Hartmann C., Bartels G., Gehlhaar C., Holtkamp N., von Deimling A. (2005). PIK3CA Mutations in Glioblastoma Multiforme. Acta Neuropathol..

[133] Knobbe C.B., Trampe-Kieslich A., Reifenberger G. (2005). Genetic Alteration and Expression of the Phosphoinositol-3-Kinase/Akt Pathway Genes PIK3CA and PIKE in Human Glioblastomas. Neuropathol. Appl. Neurobiol..

[134] Wang Q., Wang H.-D., Niu W., Pan H. (2021). New Molecular Prognostic Factors of Adult Diffuse Lower-Grade Gliomas in Post-2016 Molecular Era: A Retrospective Analysis from Single Center. Br. J. Neurosurg..

[135] Mueller W., Mizoguchi M., Silen E., D’Amore K., Nutt C.L., Louis D.N. (2005). Mutations of the PIK3CA Gene Are Rare in Human Glioblastoma. Acta Neuropathol..

[136] Saadeh F.S., Morsi R.Z., El-Kurdi A., Nemer G., Mahfouz R., Charafeddine M., Khoury J., Najjar M.W., Khoueiry P., Assi H.I. (2020). Correlation of Genetic Alterations by Whole-Exome Sequencing with Clinical Outcomes of Glioblastoma Patients from the Lebanese Population. PLoS ONE.

[137] Hartmann C., Devermann L., Gehlhaar C., Holtkamp N., von Deimling A. (2006). PIK3CA Mutations in Oligodendroglial Tumours. Neuropathol. Appl. Neurobiol..

[138] The Cancer Genome Atlas Research Network (2015). Comprehensive, Integrative Genomic Analysis of Diffuse Lower-Grade Gliomas. N. Engl. J. Med..

[139] Dono A., Alfaro-Munoz K., Yan Y., Lopez-Garcia C.A., Soomro Z., Williford G., Takayasu T., Robell L., Majd N.K., de Groot J. (2022). Molecular, Histological, and Clinical Characteristics of Oligodendrogliomas: A Multi-Institutional Retrospective Study. Neurosurgery.

[140] Baker C.L., Vaughn C.P., Samowitz W.S. (2012). A PIK3CA Pyrosequencing-Based Assay That Excludes Pseudogene Interference. J. Mol. Diagn..

[141] Ang D., O’Gara R., Schilling A., Beadling C., Warrick A., Troxell M.L., Corless C.L. (2013). Novel Method for PIK3CA Mutation Analysis: Locked Nucleic Acid-PCR Sequencing. J. Mol. Diagn..

[142] McNeill R.S., Stroobant E.E., Smithberger E., Canoutas D.A., Butler M.K., Shelton A.K., Patel S.D., Limas J.C., Skinner R., Bash R.E. (2018). PIK3CA Missense Mutations Promote Glioblastoma Pathogenesis, but Do Not Enhance Targeted PI3K Inhibition. PLoS ONE.

[143] Tateishi K., Nakamura T., Juratli T.A., Williams E.A., Matsushita Y., Miyake S., Nishi M., Miller J.J., Tummala S.S., Fink A.L. (2019). PI3K/AKT/MTOR Pathway Alterations Promote Malignant Progression and Xenograft Formation in Oligodendroglial Tumors. Clin. Cancer Res..

[144] Yu K., Lin C.-C.J., Hatcher A., Lozzi B., Kong K., Huang-Hobbs E., Cheng Y.-T., Beechar V.B., Zhu W., Zhang Y. (2020). PIK3CA Variants Selectively Initiate Brain Hyperactivity during Gliomagenesis. Nature.

[145] Alqahtani A., Ayesh H.S.K., Halawani H. (2020). PIK3CA Gene Mutations in Solid Malignancies: Association with Clinicopathological Parameters and Prognosis. Cancers.

[146] Houghton P.J. (2010). Everolimus. Clin. Cancer Res..

[147] Kwitkowski V.E., Prowell T.M., Ibrahim A., Farrell A.T., Justice R., Mitchell S.S., Sridhara R., Pazdur R. (2010). FDA Approval Summary: Temsirolimus as Treatment for Advanced Renal Cell Carcinoma. Oncologist.

[148] Shirley M. (2024). Capivasertib: First Approval. Drugs.

[149] Dhillon S., Keam S.J. (2021). Umbralisib: First Approval. Drugs.

[150] Markham A. (2014). Idelalisib: First Global Approval. Drugs.

[151] Markham A. (2017). Copanlisib: First Global Approval. Drugs.

[152] Blair H.A. (2018). Duvelisib: First Global Approval. Drugs.

[153] Markham A. (2019). Alpelisib: First Global Approval. Drugs.

[154] Blair H.A. (2025). Inavolisib: First Approval. Drugs.

[155] Banerjee T., Kim M.S., Haslam A., Prasad V. (2023). Clinical Trials Portfolio and Regulatory History of Idelalisib in Indolent Non-Hodgkin Lymphoma: A Systematic Review and Meta-Analysis. JAMA Intern. Med..

[156] Benjamin D.J., Prasad V. (2022). PI3K Inhibitors in Haematological Malignancies. Lancet Oncol..

[157] Skånland S.S., Okkenhaug K., Davids M.S. (2024). PI3K Inhibitors in Hematology: When One Door Closes…. Clin. Cancer Res..

[158] André F., Ciruelos E., Rubovszky G., Campone M., Loibl S., Rugo H.S., Iwata H., Conte P., Mayer I.A., Kaufman B. (2019). Alpelisib for PIK3CA-Mutated, Hormone Receptor-Positive Advanced Breast Cancer. N. Engl. J. Med..

[159] André F., Ciruelos E.M., Juric D., Loibl S., Campone M., Mayer I.A., Rubovszky G., Yamashita T., Kaufman B., Lu Y.S. (2021). Alpelisib plus Fulvestrant for PIK3CA-Mutated, Hormone Receptor-Positive, Human Epidermal Growth Factor Receptor-2–Negative Advanced Breast Cancer: Final Overall Survival Results from SOLAR-1. Ann. Oncol..

[160] Savas P., Lo L.L., Luen S.J., Blackley E.F., Callahan J., Moodie K., van Geelen C.T., Ko Y.A., Weng C.F., Wein L. (2022). Alpelisib Monotherapy for PI3K-Altered, Pretreated Advanced Breast Cancer: A Phase II Study. Cancer Discov..

[161] Vasan N., Razavi P., Johnson J.L., Shao H., Shah H., Antoine A., Ladewig E., Gorelick A., Lin T.-Y., Toska E. (2019). Double PIK3CA Mutations in Cis Increase Oncogenicity and Sensitivity to PI3Kα Inhibitors. Science.

[162] Xie S., Ni J., McFaline-Figueroa J.R., Wang Y., Bronson R.T., Ligon K.L., Wen P.Y., Roberts T.M., Zhao J.J. (2020). Divergent Roles of PI3K Isoforms in PTEN-Deficient Glioblastomas. Cell Rep..

[163] Eckerdt F.D., Bell J.B., Gonzalez C., Oh M.S., Perez R.E., Mazewski C., Fischietti M., Goldman S., Nakano I., Platanias L.C. (2020). Combined PI3Kα-MTOR Targeting of Glioma Stem Cells. Sci. Rep..

[164] Passarelli A., Carbone V., Pignata S., Mazzeo R., Lorusso D., Scambia G., Canova S., Di Palma T., Tasca G., Mantiero M. (2024). Alpelisib for PIK3CA-Mutated Advanced Gynecological Cancers: First Clues of Clinical Activity. Gynecol. Oncol..

[165] Bogani G., Chiappa V., Bini M., Ronzulli D., Indini A., Conca E., Raspagliesi F. (2023). BYL719 (Alpelisib) for the Treatment of PIK3CA-Mutated, Recurrent/Advanced Cervical Cancer. Tumori.

[166] Wei Y., Lin S., Zhi W., Chu T., Liu B., Peng T., Xu M., Ding W., Cao C., Wu P. (2023). Genomic Analysis of Cervical Carcinoma Identifies Alpelisib as a Therapeutic Option for PIK3CA-Mutant Cervical Carcinoma via the PI3K/AKT Pathway. J. Med. Virol..

[167] Xu H., Chen K., Shang R., Chen X., Zhang Y., Song X., Evert M., Zhong S., Li B., Calvisi D.F. (2021). Alpelisib Combination Treatment as Novel Targeted Therapy against Hepatocellular Carcinoma. Cell Death Dis..

[168] Kim K.H., Hwang S., Kim M.K., Park K.-U., Yun T., Lee K.-W., Kim J.H., Keam B., Cho B.C., Oh S.Y. (2025). Differential Efficacy of Alpelisib by PIK3CA Mutation Site in Head and Neck Squamous Cell Carcinoma: An Analysis from the KCSG HN 15-16 TRIUMPH Trial. Cancer Res. Treat..

[169] Razak A.R.A., Wang H.M., Chang J.Y., Ahn M.J., Munster P., Blumenschein G., Solomon B., Lim D.W.T., Hong R.L., Pfister D. (2023). A Phase 1b/2 Study of Alpelisib in Combination with Cetuximab in Patients with Recurrent or Metastatic Head and Neck Squamous Cell Carcinoma. Target. Oncol..

[170] Jin N., Keam B., Cho J., Lee M.J., Kim H.R., Torosyan H., Jura N., Ng P.K.S., Mills G.B., Li H. (2021). Therapeutic Implications of Activating Noncanonical PIK3CA Mutations in Head and Neck Squamous Cell Carcinoma. J. Clin. Investig..

[171] García-Carracedo D., Cai Y., Qiu W., Saeki K., Friedman R.A., Lee A., Li Y., Goldberg E.M., Stratikopoulos E.E., Parsons R. (2020). PIK3CA and P53 Mutations Promote 4NQO-Initated Head and Neck Tumor Progression and Metastasis in Mice. Mol. Cancer Res..

[172] Shi R., Li M., Raghavan V., Tam S., Cabanero M., Pham N.-A., Shepherd F.A., Moghal N., Tsao M.-S. (2018). Targeting the CDK4/6-Rb Pathway Enhances Response to PI3K Inhibition in PIK3CA-Mutant Lung Squamous Cell Carcinoma. Clin. Cancer Res..

[173] Baselga J., Im S.-A., Iwata H., Cortés J., De Laurentiis M., Jiang Z., Arteaga C.L., Jonat W., Clemons M., Ito Y. (2017). Buparlisib plus Fulvestrant versus Placebo plus Fulvestrant in Postmenopausal, Hormone Receptor-Positive, HER2-Negative, Advanced Breast Cancer (BELLE-2): A Randomised, Double-Blind, Placebo-Controlled, Phase 3 Trial. Lancet Oncol..

[174] Welt A., Wiesweg M., Theurer S., Abenhardt W., Groschek M., Müller L., Schröder J., Tewes M., Chiabudini M., Potthoff K. (2020). Buparlisib in Combination with Tamoxifen in Pretreated Patients with Hormone Receptor-Positive, HER2-Negative Advanced Breast Cancer Molecularly Stratified for PIK3CA Mutations and Loss of PTEN Expression. Cancer Med..

[175] Garrido-Castro A.C., Saura C., Barroso-Sousa R., Guo H., Ciruelos E., Bermejo B., Gavilá J., Serra V., Prat A., Paré L. (2020). Phase 2 Study of Buparlisib (BKM120), a Pan-Class I PI3K Inhibitor, in Patients with Metastatic Triple-Negative Breast Cancer. Breast Cancer Res..

[176] Kojima T., Kato K., Hara H., Takahashi S., Muro K., Nishina T., Wakabayashi M., Nomura S., Sato A., Ohtsu A. (2022). Phase II Study of BKM120 in Patients with Advanced Esophageal Squamous Cell Carcinoma (EPOC1303). Esophagus.

[177] Wen P.Y., Touat M., Alexander B.M., Mellinghoff I.K., Ramkissoon S., McCluskey C.S., Pelton K., Haidar S., Basu S.S., Gaffey S.C. (2019). Buparlisib in Patients With Recurrent Glioblastoma Harboring Phosphatidylinositol 3-Kinase Pathway Activation: An Open-Label, Multicenter, Multi-Arm, Phase II Trial. J. Clin. Oncol..

[178] Rosenthal M., Clement P.M., Campone M., Gil-Gil M.J., DeGroot J., Chinot O., Idbaih A., Gan H., Raizer J., Wen P.Y. (2020). Buparlisib plus Carboplatin or Lomustine in Patients with Recurrent Glioblastoma: A Phase Ib/II, Open-Label, Multicentre, Randomised Study. ESMO Open.

[179] Zhang S., Peng X., Li X., Liu H., Zhao B., Elkabets M., Liu Y., Wang W., Wang R., Zhong Y. (2021). BKM120 Sensitizes Glioblastoma to the PARP Inhibitor Rucaparib by Suppressing Homologous Recombination Repair. Cell Death Dis..

[180] Kim H.R., Kang H.N., Yun M.R., Ju K.Y., Choi J.W., Jung D.M., Pyo K.H., Hong M.H., Ahn M.J., Sun J.M. (2020). Mouse–Human Co-Clinical Trials Demonstrate Superior Anti-Tumour Effects of Buparlisib (BKM120) and Cetuximab Combination in Squamous Cell Carcinoma of Head and Neck. Br. J. Cancer.

[181] Soulières D., Faivre S., Mesía R., Remenár É., Li S.H., Karpenko A., Dechaphunkul A., Ochsenreither S., Kiss L.A., Lin J.C. (2017). Buparlisib and Paclitaxel in Patients with Platinum-Pretreated Recurrent or Metastatic Squamous Cell Carcinoma of the Head and Neck (BERIL-1): A Randomised, Double-Blind, Placebo-Controlled Phase 2 Trial. Lancet Oncol..

[182] Soulières D., Licitra L., Mesía R., Remenar E., Li S.H., Karpenko A., Chol M., Wang Y.A., Solovieff N., Bourdeau L. (2018). Molecular Alterations and Buparlisib Efficacy in Patients with Squamous Cell Carcinoma of the Head and Neck: Biomarker Analysis from BERIL-1. Clin. Cancer Res..

[183] Turner N.C., Im S.-A., Saura C., Juric D., Loibl S., Kalinsky K., Schmid P., Loi S., Sunpaweravong P., Musolino A. (2024). Inavolisib-Based Therapy in PIK3CA-Mutated Advanced Breast Cancer. N. Engl. J. Med..

[184] Bedard P.L., Jhaveri K.L., Accordino M.K., Cervantes P.A., Gambardella V., Hamilton E., Italiano P.A., Kalinsky P.K., Krop P.I.E., Oliveira M. (2025). Inavolisib plus Letrozole or Fulvestrant in PIK3CA-Mutated, Hormone Receptor-Positive, HER2-Negative Advanced or Metastatic Breast Cancer (GO39374): An Open-Label, Multicentre, Dose-Escalation and Dose-Expansion Phase 1/1b Study. Eur. J. Cancer.

[185] Song K.W., Edgar K.A., Hanan E.J., Hafner M., Oeh J., Merchant M., Sampath D., Nannini M.A., Hong R., Phu L. (2022). RTK-Dependent Inducible Degradation of Mutant PI3Kα Drives GDC-0077 (Inavolisib) Efficacy. Cancer Discov..

[186] Wen P.Y., Frederick De Groot J., Battiste J., Goldlust S.A., Garner J.S., Friend J., Simpson J.A., Damek D., Olivero A., Cloughesy T.F. (2022). Paxalisib in Patients with Newly Diagnosed Glioblastoma with Unmethylated MGMT Promoter Status: Final Phase 2 Study Results. J. Clin. Oncol..

[187] Guo T., Wu C., Zhang J., Yu J., Li G., Jiang H., Zhang X., Yu R., Liu X. (2023). Dual Blockade of EGFR and PI3K Signaling Pathways Offers a Therapeutic Strategy for Glioblastoma. Cell Commun. Signal.

[188] Heffron T.P., Ndubaku C.O., Salphati L., Alicke B., Cheong J., Drobnick J., Edgar K., Gould S.E., Lee L.B., Lesnick J.D. (2016). Discovery of Clinical Development Candidate GDC-0084, a Brain Penetrant Inhibitor of PI3K and MTOR. ACS Med. Chem. Lett..

[189] Salphati L., Alicke B., Heffron T.P., Shahidi-Latham S., Nishimura M., Cao T., Carano R.A., Cheong J., Greve J., Koeppen H. (2016). Brain Distribution and Efficacy of the Brain Penetrant PI3K Inhibitor GDC-0084 in Orthotopic Mouse Models of Human Glioblastoma. Drug Metab. Disp..

[190] Shi F., Guo H., Zhang R., Liu H., Wu L., Wu Q., Liu J., Liu T., Zhang Q. (2017). The PI3K Inhibitor GDC-0941 Enhances Radiosensitization and Reduces Chemoresistance to Temozolomide in GBM Cell Lines. Neuroscience.

[191] Zumsteg Z.S., Morse N., Krigsfeld G., Gupta G., Higginson D.S., Lee N.Y., Morris L., Ganly I., Shiao S.L., Powell S.N. (2016). Taselisib (GDC-0032), a Potent β-Sparing Small Molecule Inhibitor of PI3K, Radiosensitizes Head and Neck Squamous Carcinomas Containing Activating PIK3CA Alterations. Clin. Cancer Res..

[192] Wirtz E.D., Hoshino D., Maldonado A.T., Tyson D.R., Weaver A.M. (2015). Response of Head and Neck Squamous Cell Carcinoma Cells Carrying PIK3CA Mutations to Selected Targeted Therapies. JAMA Otolaryngol. Head Neck Surg..

[193] Varkaris A., Pazolli E., Gunaydin H., Wang Q., Pierce L., Boezio A.A., Bulku A., Dipietro L., Fridrich C., Frost A. (2024). Discovery and Clinical Proof-of-Concept of RLY-2608, a First-in-Class Mutant-Selective Allosteric PI3Kα Inhibitor That Decouples Antitumor Activity from Hyperinsulinemia. Cancer Discov..

[194] Dent S., Cortés J., Im Y.H., Diéras V., Harbeck N., Krop I.E., Wilson T.R., Cui N., Schimmoller F., Hsu J.Y. (2021). Phase III Randomized Study of Taselisib or Placebo with Fulvestrant in Estrogen Receptor-Positive, PIK3CA-Mutant, HER2-Negative, Advanced Breast Cancer: The SANDPIPER Trial. Ann. Oncol..

[195] Dickler M.N., Saura C., Richards D.A., Krop I.E., Cervantes A., Bedard P.L., Patel M.R., Pusztai L., Oliveira M., Cardenas A.K. (2018). Phase II Study of Taselisib (GDC-0032) in Combination with Fulvestrant in Patients with HER2-Negative, Hormone Receptor–Positive Advanced Breast Cancer. Clin. Cancer Res..

[196] Grinshpun A., Ren S., Graham N., DeMeo M.K., Wrabel E., Carter J., Tayob N., Pereslete A., Hamilton E., Juric D. (2024). Phase Ib Dose-Escalation Trial of Taselisib (GDC-0032) in Combination with HER2-Directed Therapies in Patients with Advanced HER2+ Breast Cancer. ESMO Open.

[197] Langer C.J., Redman M.W., Wade J.L., Aggarwal C., Bradley J.D., Crawford J., Stella P.J., Knapp M.H., Miao J., Minichiello K. (2019). SWOG S1400B (NCT02785913), a Phase II Study of GDC-0032 (Taselisib) for Previously Treated PI3K-Positive Patients with Stage IV Squamous Cell Lung Cancer (Lung-MAP Sub-Study). J. Thorac. Oncol..

[198] Blackwell K., Burris H., Gomez P., Lynn Henry N., Isakoff S., Campana F., Gao L., Jiang J., Macé S., Tolaney S.M. (2015). Phase I/II Dose-Escalation Study of PI3K Inhibitors Pilaralisib or Voxtalisib in Combination with Letrozole in Patients with Hormone-Receptor-Positive and HER2-Negative Metastatic Breast Cancer Refractory to a Non-Steroidal Aromatase Inhibitor. Breast Cancer Res. Treat..

[199] Wen P.Y., Omuro A., Ahluwalia M.S., Fathallah-Shaykh H.M., Mohile N., Lager J.J., Laird A.D., Tang J., Jiang J., Egile C. (2015). Phase I Dose-Escalation Study of the PI3K/MTOR Inhibitor Voxtalisib (SAR245409, XL765) plus Temozolomide with or without Radiotherapy in Patients with High-Grade Glioma. Neuro Oncol..

[200] Zhao H., Chen G., Liang H. (2019). Dual PI3K/MTOR Inhibitor, XL765, Suppresses Glioblastoma Growth by Inducing ER Stress Dependent Apoptosis. Onco Targets Ther..

[201] Batsios G., Viswanath P., Subramani E., Najac C., Gillespie A.M., Santos R.D., Molloy A.R., Pieper R.O., Ronen S.M. (2019). PI3K/MTOR Inhibition of IDH1 Mutant Glioma Leads to Reduced 2HG Production That Is Associated with Increased Survival. Sci. Rep..

[202] Arend R.C., Davis A.M., Chimiczewski P., O’Malley D.M., Provencher D., Vergote I., Ghamande S., Birrer M.J. (2020). EMR 20006-012: A Phase II Randomized Double-Blind Placebo Controlled Trial Comparing the Combination of Pimasertib (MEK Inhibitor) with SAR245409 (PI3K Inhibitor) to Pimasertib Alone in Patients with Previously Treated Unresectable Borderline or Low Grade Ovarian Cancer. Gynecol. Oncol..

[203] Inaba K., Oda K., Ikeda Y., Sone K., Miyasaka A., Kashiyama T., Fukuda T., Uehara Y., Arimoto T., Kuramoto H. (2015). Antitumor Activity of a Combination of Dual PI3K/MTOR Inhibitor SAR245409 and Selective MEK1/2 Inhibitor Pimasertib in Endometrial Carcinomas. Gynecol. Oncol..

[204] Gravina G.L., Mancini A., Scarsella L., Colapietro A., Jitariuc A., Vitale F., Marampon F., Ricevuto E., Festuccia C. (2016). Dual PI3K/MTOR Inhibitor, XL765 (SAR245409), Shows Superior Effects to Sole PI3K [XL147 (SAR245408)] or MTOR [Rapamycin] Inhibition in Prostate Cancer Cell Models. Tumor Biol..

[205] Yi Z., Ma F., Liu B., Guan X., Li L., Li C., Qian H., Xu B. (2019). Everolimus in Hormone Receptor-Positive Metastatic Breast Cancer: PIK3CA Mutation H1047R Was a Potential Efficacy Biomarker in a Retrospective Study. BMC Cancer.

[206] Varkaris A., Fece de la Cruz F., Martin E.E., Norden B.L., Chevalier N., Kehlmann A.M., Leshchiner I., Barnes H., Ehnstrom S., Stavridi A.M. (2024). Allosteric PI3Kα Inhibition Overcomes On-Target Resistance to Orthosteric Inhibitors Mediated by Secondary PIK3CA Mutations. Cancer Discov..

[207] Moynahan M.E., Chen D., He W., Sung P., Samoila A., You D., Bhatt T., Patel P., Ringeisen F., Hortobagyi G.N. (2017). Correlation between PIK3CA Mutations in Cell-Free DNA and Everolimus Efficacy in HR+, HER2-Advanced Breast Cancer: Results from BOLERO-2. Br. J. Cancer.

[208] De Santis M.C., Gulluni F., Campa C.C., Martini M., Hirsch E. (2019). Targeting PI3K Signaling in Cancer: Challenges and Advances. BBA Rev. Cancer.

[209] Furet P., Guagnano V., Fairhurst R.A., Imbach-Weese P., Bruce I., Knapp M., Fritsch C., Blasco F., Blanz J., Aichholz R. (2013). Discovery of NVP-BYL719 a Potent and Selective Phosphatidylinositol-3 Kinase Alpha Inhibitor Selected for Clinical Evaluation. Bioorg. Med. Chem. Lett..

[210] Chakrabarty A., Sánchez V., Kuba M.G., Rinehart C., Arteaga C.L. (2012). Feedback Upregulation of HER3 (ErbB3) Expression and Activity Attenuates Antitumor Effect of PI3K Inhibitors. Proc. Natl. Acad. Sci. USA.

[211] Serra V., Scaltriti M., Prudkin L., Eichhorn P.J.A., Ibrahim Y.H., Chandarlapaty S., Markman B., Rodriguez O., Guzman M., Rodriguez S. (2011). PI3K Inhibition Results in Enhanced HER Signaling and Acquired ERK Dependency in HER2-Overexpressing Breast Cancer. Oncogene.

[212] Hopkins B.D., Pauli C., Xing D., Wang D.G., Li X., Wu D., Amadiume S.C., Goncalves M.D., Hodakoski C., Lundquist M.R. (2018). Suppression of Insulin Feedback Enhances the Efficacy of PI3K Inhibitors. Nature.

[213] Le X., Antony R., Razavi P., Treacy D.J., Luo F., Ghandi M., Castel P., Scaltriti M., Baselga J., Garraway L.A. (2016). Systematic Functional Characterization of Resistance to PI3K Inhibition in Breast Cancer. Cancer Discov..

[214] Juric D., Castel P., Griffith M., Griffith O.L., Won H.H., Ellis H., Ebbesen S.H., Ainscough B.J., Ramu A., Iyer G. (2015). Convergent Loss of PTEN Leads to Clinical Resistance to a PI(3)Kα Inhibitor. Nature.

[215] Elkabets M., Vora S., Juric D., Morse N., Mino-Kenudson M., Muranen T., Tao J., Campos A.B., Rodon J., Ibrahim Y.H. (2013). MTORC1 Inhibition Is Required for Sensitivity to PI3K P110α Inhibitors in PIK3CA-Mutant Breast Cancer. Sci. Transl. Med..

[216] Schwartz S., Wongvipat J., Trigwell C.B., Hancox U., Carver B.S., Rodrik-Outmezguine V., Will M., Yellen P., de Stanchina E., Baselga J. (2015). Feedback Suppression of PI3Kα Signaling in PTEN-Mutated Tumors Is Relieved by Selective Inhibition of PI3Kβ. Cancer Cell.

[217] Costa C., Ebi H., Martini M., Beausoleil S.A., Faber A.C., Jakubik C.T., Huang A., Wang Y., Nishtala M., Hall B. (2015). Measurement of PIP3 Levels Reveals an Unexpected Role for P110β in Early Adaptive Responses to P110α-Specific Inhibitors in Luminal Breast Cancer. Cancer Cell.

[218] Liu Y., Cui B., Qiao Y., Zhang Y., Tian Y., Jiang J., Ma D., Kong B. (2011). Phosphoinositide-3-Kinase Inhibition Enhances Radiosensitization of Cervical Cancer in Vivo. Int. J. Gynecol. Cancer.

[219] Zhang T., Cui G.-B., Zhang J., Zhang F., Zhou Y.-A., Jian T., Li X.-F. (2010). Inhibition of PI3 Kinases Enhances the Sensitivity of Non-Small Cell Lung Cancer Cells to Ionizing Radiation. Oncol. Rep..

[220] The Cancer Genome Atlas Research Network (2008). Comprehensive Genomic Characterization Defines Human Glioblastoma Genes and Core Pathways. Nature.

[221] Srividya M.R., Thota B., Shailaja B.C., Arivazhagan A., Thennarasu K., Chandramouli B.A., Hegde A.S., Santosh V. (2011). Homozygous 10q23/PTEN Deletion and Its Impact on Outcome in Glioblastoma: A Prospective Translational Study on a Uniformly Treated Cohort of Adult Patients. Neuropathology.

[222] Wee S., Wiederschain D., Maira S.-M., Loo A., Miller C., DeBeaumont R., Stegmeier F., Yao Y.-M., Lengauer C. (2008). PTEN-Deficient Cancers Depend on PIK3CB. Proc. Natl. Acad. Sci. USA.

